# Color-tunable bioluminescence imaging portfolio for cell imaging

**DOI:** 10.1038/s41598-021-81430-1

**Published:** 2021-01-26

**Authors:** Shota Tamaki, Nobuo Kitada, Masahiro Kiyama, Rika Fujii, Takashi Hirano, Sung Bae Kim, Shojiro Maki

**Affiliations:** 1 Department of Engineering Science, Graduate School of Informatics and Engineering, The University of Electro-Communications, Chofu, Tokyo 182-8585 Japan; 2 Research Institute for Environmental Management Technology, National Institute of Advanced Industrial Science and Technology (AIST), 16-1 Onogawa, Tsukuba, 305-8569 Japan

**Keywords:** Biological techniques, Biotechnology, Chemical biology, Molecular biology, Chemistry, Optics and photonics

## Abstract

The present study describes a color-tunable imaging portfolio together with twelve novel coelenterazine (CTZ) analogues. The three groups of CTZ analogues create diverse hues of bioluminescence (BL) ranging from blue to far red with marine luciferases. We found that the hue completes the whole color palette in the visible region and shows red-shifted BL with a marine luciferase: for example, *Renilla* luciferase 8 (RLuc8) and Artificial Luciferase 16 (ALuc16) show 187 nm- and 105 nm-redshifted spectra, respectively, by simply replacing the substrate CTZ with **1d**. The optical properties of the new CTZ analogues were investigated such as the kinetic parameters, dose dependency, and luciferase specificity. The 2-series CTZ analogues interestingly have specificity to ALucs and are completely dark with RLuc derivatives, and **3d** is highly specific to only NanoLuc. We further determined the theoretical background of the red-shifted BL maximum wavelengths (*λ*_BL_) values according to the extended π conjugation of the CTZ backbone using Density Functional Theory (DFT) calculations. This color-tunable BL imaging system provides a useful multicolor imaging portfolio that efficiently images molecular events in mammalian cells.

## Introduction

Cells provoke diverse intracellular signal transductions in response to a myriad of stimuli from the surrounding environment^1^. As cellular systems are such dynamical entities, multiplex imaging is a plausible modality for spying and visualizing such molecular events in cells. To date, bioluminescence (BL) has been broadly utilized for imaging diverse molecular events in the complex context of living subjects^2^. However, conventional BL systems have mostly depended on a limited color palette and narrow choices of luciferases such as firefly luciferase (FLuc) and *Renilla* luciferase (RLuc)^3^. The limited color palette at shorter wavelengths commonly suffers from severe attenuation by hemoglobin; hence red-shifted BL is more appropriate for in vivo imaging of signals from deep physiological tissues^4^. This region ranging from 600 to 900 nm is especially called an “optical window”.

To address this limitation, many researchers have focused on expending the optical repertories toward red and near infrared (NIR) region. Thanks to the efforts, a couple of excellent NIR imaging systems have been developed. Yao et al. developed a multicolor imaging system with a beetle luciferase and a π-Extended Luciferin^5^. We also developed a bioluminescence imaging (BLI) system with Akalumine and FLuc variants, and applied it to even few cells in animal models^6^, where the maximal optical intensity (*λ*_max_) reaches 650 nm with AkaLuc^7^. Further, we developed dye-conjugated CTZ analogues^8^ and a CTZ analogue emitting NIR BL for through-bond energy transfer (TBET)-based imaging modalities^9^ for far-red and NIR imaging.

In contrast to the success with red-shifted substrates and mutated luciferases, the color palette of marine luciferases still has been confined mostly in blue and green region. Among marine luciferases, RLuc and its derivatives have been mostly used in conventional BL imaging systems. Nevertheless, even RLuc derivatives merely reached 545 nm at the *λ*_max_ by reacting to the unstable CTZ analogue, CTZ*v*^10^. Recently, Kim et al. developed Artificial Luciferases (ALuc), however, they emit BL only in the greenish-blue region, thus having an incomplete color palette with respect to multicolor imaging modality.

NanoLuc has been utilized in various BLI systems, one of which maximally luminesces at 583 nm through combining red-shifted coelenterazine (CTZ) analogues and a fusion protein of NanoLuc named “Antares2”^11^. It is known that even NanoLuc alone has potential to emit red-shifted BL with Furimazine analogues^12^. NanoLuc was further modified for bioluminescence resonance energy transfer (BRET)-based multicolor BLI by chemically labeling it with either SNAP‐tag or HaloTag7^13^.

We previously accomplished an NIR imaging system^4^, which surprisingly achieved a ca. 300 nm *blue-to-NIR* shift of BRET by combining a novel CTZ analogue, named “Bottle Blue (BBlue)”, with an infrared fluorescent protein (iRFP)-linked RLuc8.6-535SG (iRFP-RLuc8.6-535SG) fusion protein as a probe. The *λ*_max_ reached 715 nm after BRET.

**Figure 1:**
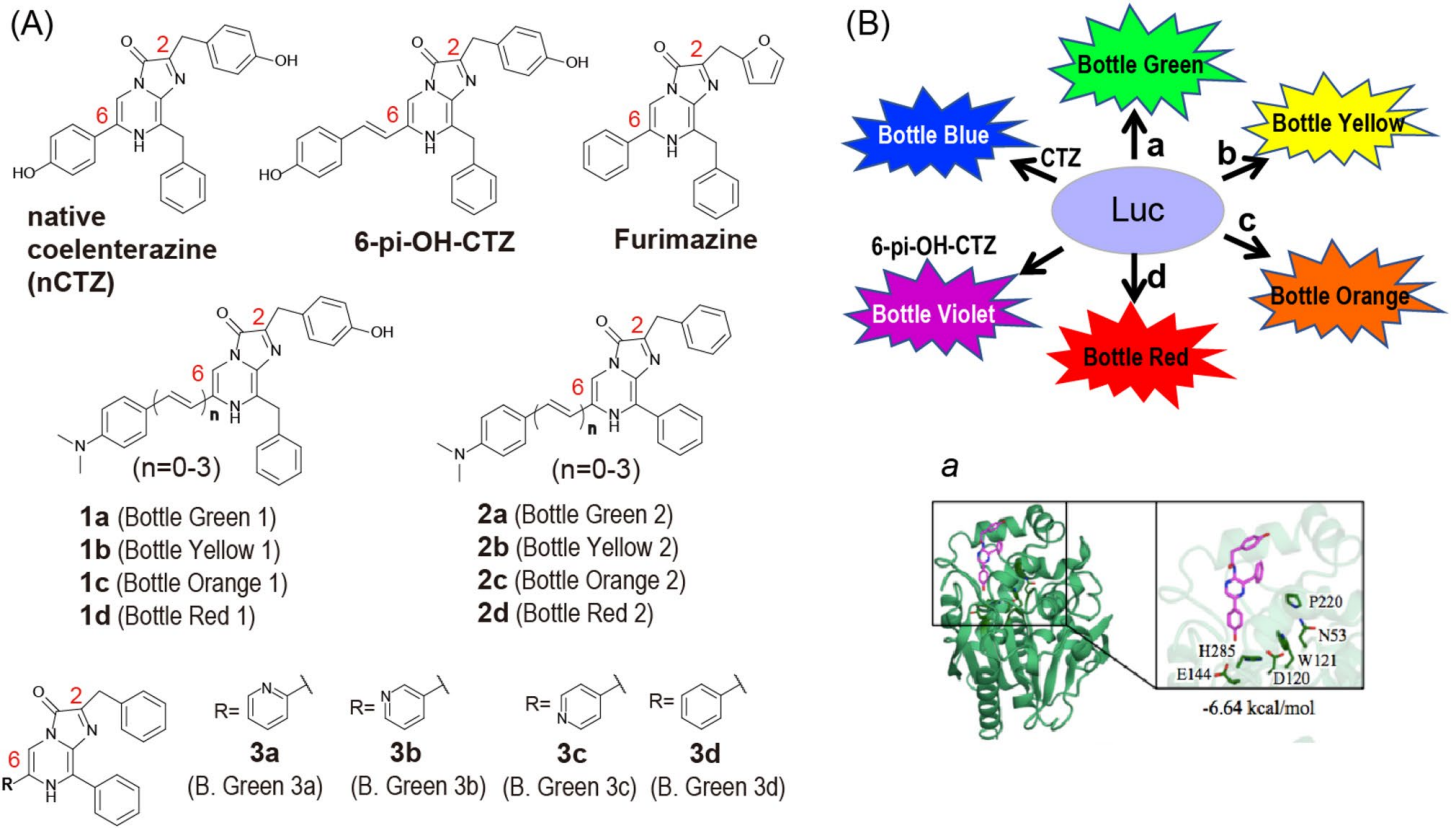
(**A**) Chemical structures of CTZ analogues for marine luciferases. The 12 luciferins were categorized into three groups according to the chemical structures: 1, 2, and 3. B. Green means Bottle Green. (**B**) Schematic diagram illustrating a color-tuneable BL imaging system, where the alphabets **a**, **b**, **c**, and **d** symbolize respective CTZ analogues. Inset ***a*** shows the binding model between RLuc8 and coelenteramide.

In the present study, we synthesized nine novel CTZ analogues together with three known analogues (**1a**^14^, **3c**^15,16^, **3d**^11,15^). They were categorized into three groups, which create diverse hues of BL colors ranging from blue to far red with marine luciferases, where any visible colors may be generated by simply mixing a marine luciferase with one of the CTZ analogues (Figs. 1, S8 and S9). The synthesis procedures were described in Figs. S8 and S9 in detail. Initially, we characterized the optical properties of the newly synthesized CTZ analogues such as the kinetic parameters, dose dependency, and luciferase specificity. We further investigated the theoretical background of the red-shifted BL maximum wavelengths (*λ*_BL_) values according to the extended π conjugation at the C6 position of the CTZ backbone using Density Functional Theory (DFT) and Time-Dependent Density Functional Theory (TD-DFT) calculations.

The present color-tunable BL imaging system is an important addition to the molecular imaging studies in vitro and in vivo.

## Results and discussion

### Synthesis of CTZ analogues

The basic molecular designs in Fig. 1 were inspired by our precedent studies with luciferin analogues such as Akalumine^17^ and 6-pi-OH-CTZ^18^: (i) The first idea is to exert the significant redshifts of BL by replacing the benzothiazole backbone of D-Luciferin with an extended π conjugation^7,17^; and (ii) the second idea is to develop the luciferase specificity through the π conjugation at the C6 position of the CTZ backbone^18^. Herein, we introduced the appendages with various lengths of the π conjugation at the C6 position of the CTZ backbone, inspired by these two ideas. Furthermore, we deployed a dimethylamino group at the *para* position of the phenyl moiety in the appendages at C6 that mimics the same functional group as in Akalumine. The dimethylamino group plays important roles as an electron donating group (EDG) to increase the luminescence quantum yield and modulate emission wavelength^19^. Based on all the ideas, we synthesized the CTZ analogues **1a**–**d** and **2a**–**d**. The analogues were categorized into two groups, Group 1 and Group 2, with respect to the presence or absence of a hydroxyl group (-OH) at the benzene backbone of the C2 position (Fig. 1).

Separately, we synthesized another category of the CTZ analogues, that is specific to NanoLuc (Group 3), where the phenyl group at the C6 position of Furimazine^20^ was substituted by a pyridine group, while the furan group at the C2 position was replaced with a benzene (Fig. 1).

CTZ analogues are abbreviated as “[number] plus [alphabet]” according to Rules 1 and 2, wherein the groups are represented as 1, 2, or 3 (Rule 1); and the double bind numbers at the C6 position are alphabetized as *a* (0 double bond), *b* (1 double bond), *c* (2 consecutive double bonds), and *d* (3 consecutive double bonds) (Rule 2). CTZ analogues may be called Bottles Green, Yellow, Orange, or Red according to the colors as shown in Fig. 1.

### Full color spectra of bioluminescence

**Figure 2:**
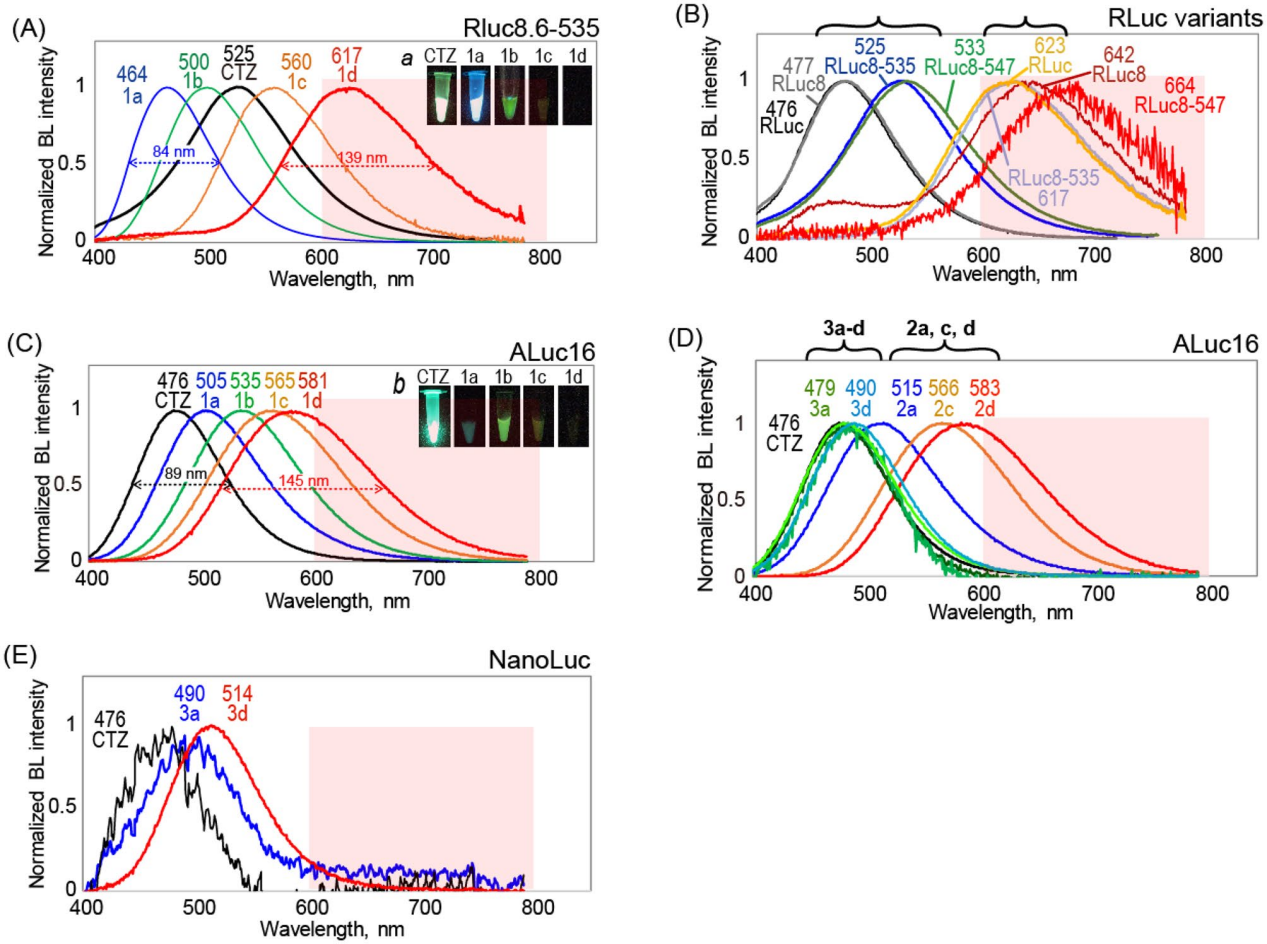
The BL spectra of the CTZ analogues **1a–d**, **2a–d**, and **3a–d** according to marine luciferases. (**A**) The BL spectra of 1-series CTZ analogues with RLuc8.6–535. Inset ***a*** shows the optical image of BL. (**B**) The BL spectra of **1d** according to various RLuc derivatives. (**C**) The BL spectra of 1-series CTZ analogues with ALuc16. Inset ***b*** shows the optical image of BL. (**D**) The BL spectra of 2- and 3-series CTZ analogues with ALuc16. (**E**) The BL spectra of **3a** and **3d** with NanoLuc. Abbreviations: CTZ, native coelenterazine; FWHM, full width half maximum; BL, bioluminescence.

The BL spectra of the CTZ analogues according to marine luciferases were obtained with a highly sensitive spectrometer that simultaneously captures photons with the entire wavelength range in one shot (AB-1850, ATTO) (Fig. 2, Figures S1–S3).

The *λ*_BL_ of RLuc variants and ALuc16 are found to be red-shifted in the descending order of **1a**, **1b**, **1c**, and **1d** (Fig. 2A,C). The similar tendency is observed with 2-series CTZ analogues. Interestingly, the full width half maximum (FWHM) is broadened in the same order (Table 1): i.e., 89 nm for nCTZ; 113 nm for **1b**; 145 nm for **1d**. The results indicate that the expansion of the π conjugation by increase of the number of double bonds at the C6 position of the CTZ skeleton is the critical determinant for the red- shifts of the BL spectra with marine luciferases. Thus, **1d** and **2d** exert the most red-shifted BL spectra in the groups.

**Table 1:** BL properties of CTZ analogues with various marine luciferases.

Comp	*λ*_BL_^a^ (nm)						RLuc8.6-535				ALuc16			
	RLuc	RLuc8	RLuc8.6 -535	RLuc8.6 -547	ALuc16	NanoLuc	Total Int. ^**b**^ (%)	Int > 600 ^**c**^ (%)	*K*_m_ (μM) ^**d**^	FWHM (nm)	Total Int (%)	Int > 600 (fold)	*K*_m_ (μM)	FWHM (nm)
**CTZ**	476	477	525	533	476	472	100	100	26.5	116	100	100	38.5	89
**1a**	478	469	464	484	505	–	**297**	38	77.2	84	111	393	–	105
**1b**	504	518	500	543	535	–	81	32	15.1	97	187	**1,297**	**7.9**	**113**
**1c**	555	567	560	599	565	–	10	21	14.3	113	53	741	26.1	132
**1d**	623	642 (451)	617	664	581	–	12	59	20.9	139	31	619	70.6	145
**2a**	–	563	–	–	515	–	0	1	–		39	192	–	116
**2b**	–	513	–	–	–	–	0	1	–		1	17	–	–
**2c**	–	498	–	–	566	–	0	1	–		**91**	**1,299**	157.3	**129**
**2d**	–	–	–	–	583	–	0	1	–		45	917	86.5	144
**3a**	501	520	500	512	479	490	0	0	–		6	21	–	96
**3b**	–	–	–	–	484	490	0	0	–		3	19	–	97
**3c**	490	520	487	496	480	449	0	0	–		46	64	68.3	96
**3d**	–	515	–	–	490	514	0	0	–		28	50	120.1	96

The table summarizes BL maximum wavelengths (*λ*_BL_), the total BL intensities (Total Int.), the intensity ratios exceeding 600 nm over the total intensities (Int > 600), Michaelis constants (*K*_m_), and full width half maximum (FWHM). Abbreviations: CTZ, native coelenterazine; RLuc, *Renilla* luciferase; RLuc8, *Renilla* luciferase 8; RLuc8.6–535, *Renilla* luciferase 8.6–535; ALuc16, Artificial luciferase 16.

^a^BL maximum wavelength. ^b^The whole BL intensities of CTZ analogues, compared to that of CTZ. ^c^The ratios of the BL intensities that is longer than 600 nm in the wavelength. ^d^The Michaelis–Menten constant.

Figure 2B highlights the red-shifted spectra of RLuc variants with **1d,** compared to CTZ. We found that all the *λ*_BL_ values of the RLuc variants with **1d** exceed 600 nm. The *λ*_BL_ values were found at 642, 617, and 664 with RLuc8, RLuc8.6–535, and RLuc8.6-547, respectively (Figs. 2, S1–S3). The most dramatic red-shifts were observed with RLuc8, whose *λ*_BL_ value shifted from 477 to 642 nm (165 nm gap) through simply replacing CTZ with **1d**. Likewise, the *λ*_BL_ value of ALuc16 was observed from 476 to 581 nm according to the substrates, whose gap was found to be ca. 105 nm between CTZ and **1d**.

The overall results may be summarized as: (i) the BL colors of marine luciferases are freely tunable from blue to far red (all the visible region) simply replacing the CTZ analogues; and (ii) the extended π conjugation is the principle ingredient for the red-shifted *λ*_BL_ values with marine luciferases. The notable structural variance between the 1- and 2-series CTZ analogues is whether a hydroxy (OH) group is possessed in the benzyl moiety at the C2 position, and whether the substituent at the C8 position is a benzyl or a phenyl group. Although this is a small structural variance, the 2-series analogues only showed negligible luminesce with all the RLuc derivatives. In contrast, the same 2-series analogues significantly emit BL with ALuc16 and preserved the red-shifted tendency according to the extended π conjugation (Fig. 2D).

This result is consistent with our previous conclusion that specifies the importance of the C2 position of the CTZ backbone in the luciferase specificity^18,21^. We previously showed that CTZh which has no OH group in the C2 position still keeps its RLuc activities^21^. However, the same OH group-deficient 2-series substrates in this study did not show considerable BL intensities with the RLuc derivatives, together with the phenyl group at the C8 position. These results suggest that the CTZ analogues are active with RLuc derivatives, together with the OH group in the benzyl moiety at the C2 position and the phenyl group at the C8 position.

In contrast to the 1- and 2-series analogues, the 3-series analogues failed to develop notably red-shifted BL with conventional marine luciferases. The overall absolute BL intensity is weak excepting the case that **3d** selectively emits significant BL with NanoLuc (Fig. 2E).

### Analysis of the red-shifts based on density functional theory

**Scheme 1:**
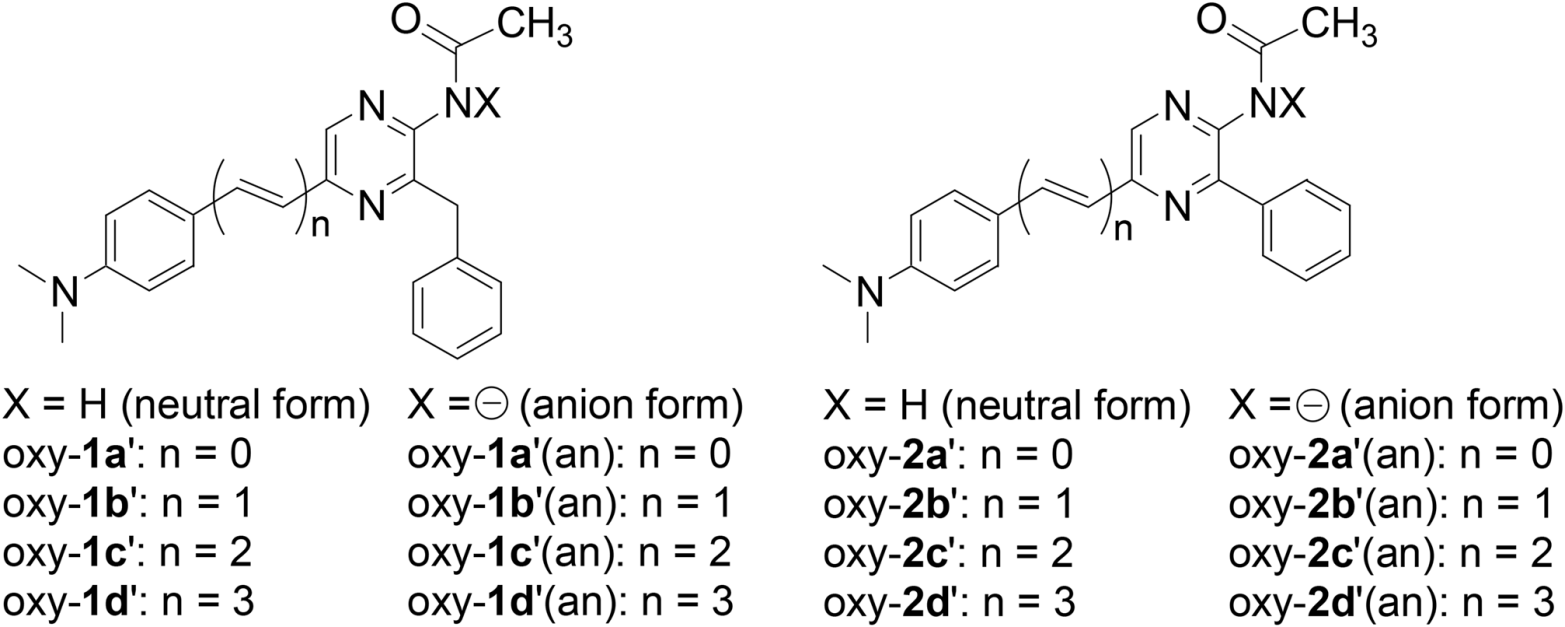
Molecular structures of acetamidopyrazine cores of the amide products for **1a**–**d** and **2a**–**d** and their amide anions.

To investigate a determinant of the *λ*_BL_ shifts by the extended π conjugation at C6 of the CTZ backbone in the 1- and 2-series analogues, we carried out Density Functional Theory (DFT) and Time-Dependent Density Functional Theory (TD-DFT) calculations of the acetamidopyrazine cores [oxy-**1a**′–**d**′ and oxy-**2a**′–**d**′ (Scheme 1)] of the amide products for **1a**-**d** and **2a**-**d** with the B3LYP/6–31 + G(d) method^22–24^ (Table 2). As the possible structures of the excited products generated by the BL reactions^25,26^, both the neutral and amide anion forms were investigated (Scheme 1). For calculations, the integral-equation formulation polarizable continuum model (IEF-PCM) approximation^27^ was also adopted with DMSO as a solvent widely employed for imidazopyrazinone chemistry^28^.

**Table 2:** Calculation data of acetamide cores oxy-**1a**′–**d**′ and oxy-**2a**′–**d**′ of the amide products from **1a**–**d** and **2a**–**d** and the corresponding amide anions with DFT and TD-DFT using B3LYP/6–31 + G(d) (IEF-PCM, DMSO).

Comp	HOMO/eV	LUMO/eV	*λ*_tr_/nm (*f*)^a^ for S_0_ → S_1_	Configuration^b^
oxy-**1a**′	− 5.37	− 1.79	395 (**0.43**)	H → L 0.69 H → L + 1 − 0.14
oxy-**1b**′	− 5.19	− 2.10	450 (**0.96**)	H → L 0.70
oxy-**1c**′	− 5.07	− 2.25	485 (**1.42**)	H → L 0.70
oxy-**1d**′	− 4.98	− 2.38	524 (**1.85**)	H → L 0.70
oxy-**2a**′	− 5.37	− 1.86	413 (**0.22**)	H → L 0.69 H → L + 1 0.12
oxy-**2b**′	− 5.20	− 2.13	459 (**0.86**)	H → L 0.69 H → L + 1 − 0.12
oxy-**2c**′	− 5.08	− 2.27	492 (**1.37**)	H → L 0.70
oxy-**2d**′	− 4.98	− 2.40	530 (**1.84**)	H → L 0.70
oxy-**1a**′(an)	− 4.94	− 1.09	372 (**0.51**)	H → L 0.68 H → L + 1 0.12
oxy-**1b**′(an)	− 4.80	− 1.50	427 (**1.21**)	H → L 0.70
oxy-**1c**′(an)	− 4.73	− 1.73	459 (**1.84**)	H → L 0.70
oxy-**1d**′(an)	− 4.69	− 1.94	497 (**2.39**)	H → L 0.70
oxy-**2a**′(an)	− 4.99	− 1.36	403 (**0.19**)	H − 1 → L − 0.10 H → L 0.70
oxy-**2b**′(an)	− 4.83	− 1.61	447 (**0.84**)	H → L 0.69 H → L + 1 0.12
oxy-**2c**′(an)	− 4.76	− 1.80	473 (**1.54**)	H → L 0.69 H → L + 1 0.13
oxy-**2d**′(an)	− 4.72	− 1.99	505 (**2.21**)	H → L 0.70

^a^Wavelengths (*λ*_tr_) estimated from the energies for S_0_ → S_1_ transitions. Oscillator strengths (*f*) are in parentheses.

^b^H − 1, H, L and L + 1 denote the HOMO − 1, HOMO, LUMO and LUMO + 1, respectively. “an” means an anionic form.

The S_0_ → S_1_ transitions of all compounds are of π,π* having the main contribution of the HOMO → LUMO configurations. The wavelengths (*λ*_tr_) estimated from the calculated transition energies can be used as the values to evaluate electronic absorption and fluorescence wavelengths of the amide products from **1a**–**d** and **2a**–**d**. The extension of the π-electronic conjugation by the ethene unit insertion effectively induces a large red-shift of the *λ*_tr_ value both in the neutral and anion forms for both the 1- and 2-series compounds. An increase in the number of the inserted ethene unit leads to decrease the HOMO–LUMO gap efficiently, resulting in a large variation of the *λ*_tr_ values. This result supports the experimental consequences that **1a**–**d** and **2a**–**d** showed the large red-shifts of the *λ*_BL_ values.

The *λ*_tr_ values of oxy-**2a**′–**d**′ and their anions are slightly red-shifted, compared with those of oxy-**1a**′–**d**′ and their anions, indicating that the phenyl groups of oxy-**2a**′–**d**′ and their anions function a little to expand π conjugation. In particular, phenyl-conjugation mainly lowers the LUMO levels of oxy-**2a**′–**d**′ and their anions compared with those of oxy-**1a**′–**d**′ and their anions, resulting in decreases of the HOMO–LUMO gaps. The observations of the *λ*_BL_ values of ALuc16 with **1a**–**d** and **2a**–**d** matches the trend of the calculation result.

Interestingly, the *λ*_tr_ values of oxy-**1a**′–**d**′ (an) and oxy-**2a**′–**d**′ (an) are 10–27 nm blue-shifted compared with those of the corresponding neutral forms, but the calculation data indicate that it is not easy to discriminate the neutral and amide anion forms of the excited products generated by the BL reactions based on the observed *λ*_BL_ values. Only oxy-**1a**′ has already been investigated its fluorescence property, to indicate that the excited oxy-**1a**′ has polarized character to show a large fluorescence solvatochromism^29^. To confirm the structures of the excited products of the BL reactions, we need to investigate the fluorescence property of the amide products further.

### Optical intensity and specificity

We further compared the absolute BL intensities of the CTZ analogues with RLuc variants (Fig. S3). The absolute BL intensities have a strong tendency to decrease as they proceed from **1a** to **1d** in the 1-series: e.g., the highest is with **1a**, whereas the lowest is with **1c** or **1d**. Upon comparison of the fold intensities, **1a** and **1b** showed 2.2- and 1.6-fold brighter than CTZ, respectively (Fig. S3), while **1c** and **1d** luminesce 0.4- and 0.6-fold levels, respectively, compared to CTZ. On the other hand, ALuc16 showed the highest BL intensity with **1b** (ca. 1.5-fold brighter than with CTZ) and then with **1a**.

**Figure 3:**
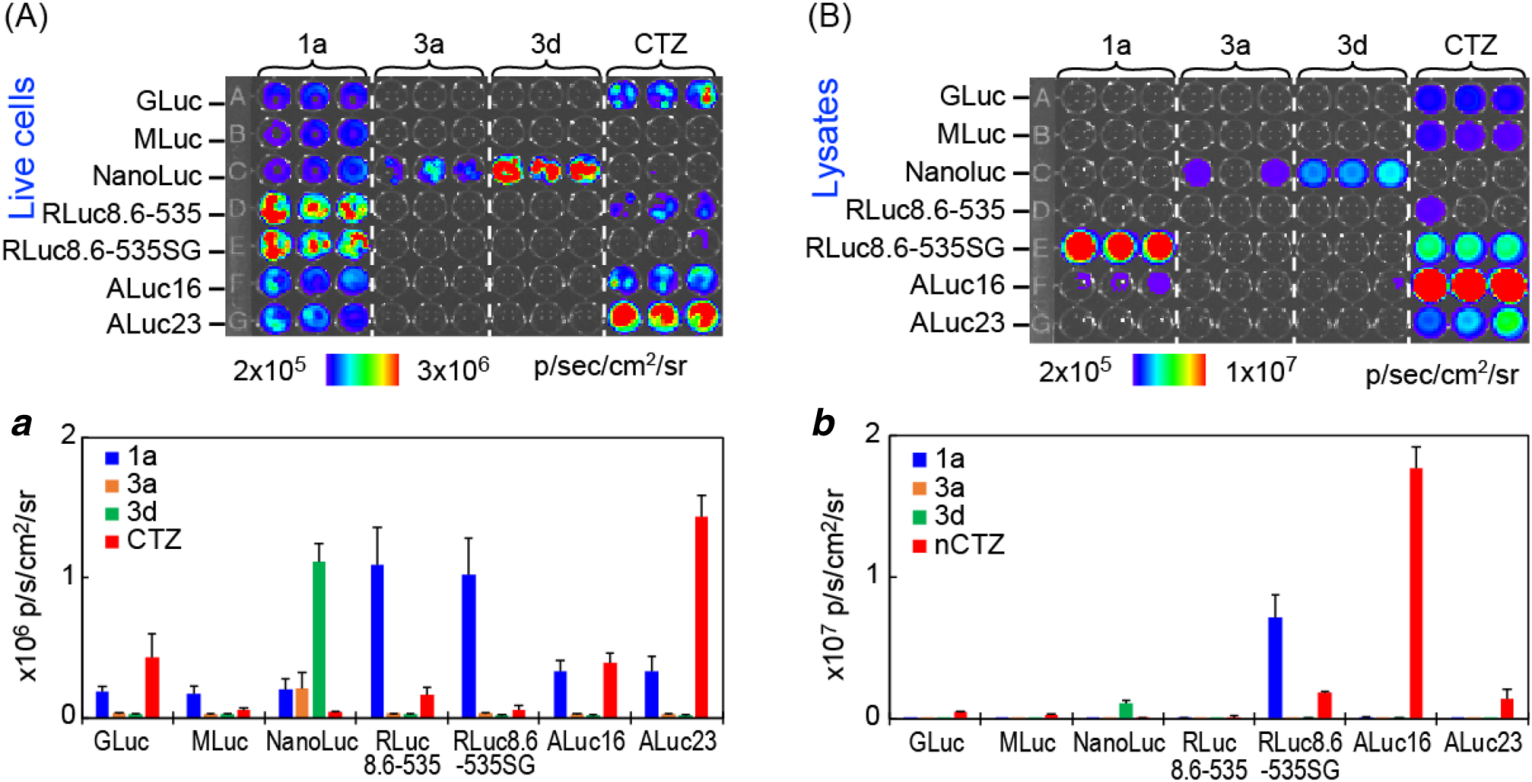
Luciferase-specific BLI of the selected CTZ analogues. (**A**) The BL image of selected CTZ analogues in live COS-7 cells. Inset ***a*** compares the BL intensities of the BL image. (**B**) The BL image of selected CTZ analogues in cell lysates. Inset ***b*** specifies the BL intensities of the BL image.

One of the most unique features of the present CTZ analogues is the luciferase specificity (Fig. 3). RLuc8.6-535SG specifically luminesces only with the 1-series CTZ analogues in both live cells and lysates, whereas ALucs are significantly bright with both 1- and 2-series CTZ analogues. Meanwhile, **3d** is highly specific only to NanoLuc. Upon comparison of the lysate with the live cell imaging, the lysates allowed better signal-to-background ratios with **1a** and CTZ.

It is interesting to compare the optical intensities of ALuc16 and ALuc23 in live cells and lysates. In live cells, ALuc23 is brighter than ALuc16. However, the BLI is reversed in the lysates. This optical discrepancy in live cells and lysates between ALuc16 and ALuc23 may be the results affected by their distinctive sensitivities to the pH and ingredients like a detergent in the lysis buffer, besides their distinctive optical kinetics.

Altogether, the results show that strong BL in blue is generated with the combinations of RLuc8.6-535—**la** (*λ*_max_: 464 nm) and RLuc—**1a** (*λ*_max_: 478 nm). This RLuc8.6–535—**la** combination is surprisingly 4.3-fold brighter than conventional RLuc8.6–535—CTZ combination. Bluish green BL is observed with RLuc8.6–535—**1b** combination (*λ*_max_: 500 nm), intensity of which is almost equivalent to RLuc8.6–535—CTZ combination.

Green BL is made with ALuc16—**1a** (*λ*_max_: 505 nm) and ALuc16—**1b** combinations (*λ*_max_: 535 nm). The ALuc16—**1b** combination is almost 1.5-fold brighter than ALuc16—CTZ combination. Yellowish green BL is generated with RLuc—**1c** (*λ*_max_: 555 nm) and ALuc16—**1c** combinations (λ_max_: 565 nm). The spectrum portion longer than 600 nm of ALuc16—**1c** combination occupies 32% over the total spectrum area. Yellow color BL is observed with ALuc16—**1d** combination (*λ*_max_: 581 nm). The 46.5% of the spectrum area is located at the region longer than 600 nm. Red BL is generated with RLuc—**1d** combination (*λ*_max_: 623 nm), where 77% of the total spectrum area is placed in the wavelength longer than 600 nm.

The results also recommend using NanoLuc—**3d** combination for exclusive specificity against the other combinations. RLuc8.6-535SG—**1a** combination is also found to show exclusive specificity each other in lysates.

### Kinetics and dose dependency

The kinetic parameters are an important determinant to evaluate the optical performance of the CTZ variants with the marine luciferases. We investigated Michaelis constant (*K*_m_) of the selected substrates based on Lineweaver–Burk equation and summarized it in Table 1. The *K*_m_ values of the 1-series substrates with RLuc8.6–535 and ALuc16 range from 7.9 to 77.2 µM. The lowest *K*_m_ value was observed with the combination of **1b** plus ALuc16. It is interpreted that **1b** has the highest binding property with ALuc16, among tested. Naturally, the **1b**—ALuc16 reaction exerted the highest BL intensity among tested. The most red-shifted substrates in each series, i.e., **1d**, **2d**, and **3d**, commonly showed relatively weak binding property with ALuc16 in the respective categories.

The substrate concentration-driven BL intensities are also determined with RLuc8.6-535 and ALuc16 (Figs. S4 and S5). It has a tendency that the higher substrate concentrations exert stronger BL intensities. The BL intensities of CTZ and **3d** start enhancing at 2.5 µM and reach to a plateau at ca. 100 µM with ALuc16. The BL intensities of **1a** start enhancing at 10 µM and keep raising even at ca. 100 µM with both RLuc8.6-535 and ALuc16. The overall results suggest that the optimal substrate concentration is ca. 100 µM with both RLuc8.6-535 and ALuc16.

## Summary

Taken together, we developed a color-tunable BL imaging portfolio creating diverse hues of visible colors ranging from 464 nm blue to 664 nm far red colors with marine luciferases, where any visible colors may be generated by mixing a marine luciferase with one of the CTZ analogues. One may specifically image diverse molecular events involving multiple marine luciferases with various colors. As some of the versatile CTZ analogues surprisingly have significant luciferase specificity, e.g., 2-series and **3d**, such CTZ analogues convince simultaneous imaging of molecular events in a single system. The present color-tunable BL imaging system is an important addition to the molecular imaging studies in vitro and in vivo as this system can easily create diverse hues of visible colors with marine luciferases, together with significant specificity.

## Experimental procedures

### Preparation of the plasmids encoding a marine luciferase

The pcDNA 3.1( +) plasmids (Invitrogen) encoding various marine luciferases were obtained from our previous studies^4,8,30,31^: The luciferases include *Renilla* luciferase (RLuc), *Renilla* luciferase 8 (RLuc8), *Renilla* luciferase 8.6-535 (RLuc8.6-535), *Renilla* luciferase 8.6-535SG (RLuc8.6-535SG), *Renilla* luciferase 8.6-547 (RLuc8.6-547), *Gaussia* luciferase (GLuc, GenBank AAG54095.1), *Metridia longa* luciferase (MLuc), NanoLuc, Artificial Luciferase 16 (ALuc16, GenBank MF817967), and Artificial Luciferase 23 (ALuc23, MF817968).

Briefly, A pMetluc2 vector encoding *Metridia longa* luciferase (MLuc) was purchased from Clontech (Mountain View, CA). The RLuc variants (RLuc8, RLuc8.6–535, and RLuc8.6-535SG) were generously gifted by Dr. Sanjiv S. Gambhir (Stanford University) or Dr. Moritoshi Sato (Tokyo University). The cDNAs encoding the other marine luciferases were custom-synthesized by Eurofins Genomics, based on the open information of the cDNA sequences in the National Center for Biotechnology Information (NCBI) database.

The cDNA fragments were ligated and subcloned in pcDNA3.1(+) vectors for mammalian cell expression. The overall sequence fidelity was confirmed with a sequencing service provided by Eurofins Genomics (Tokyo).

### Determination of bioluminescence spectra of the CTZ analogues

The BL spectra of the CTZ analogues were determined according to various luciferases (Figs. 2 and S2, S3, and S4).

The MDA-MB-231 cells derived from epithelial, human breast cancer cell line were originally cultured in 6-well microplates (Nunc) using a Dulbecco's Modified Eagle Medium (DMEM) supplemented with 10% fetal bovine serum (FBS) and 1% penicillin–streptomycin (final concentration: 100 U/mL). The cells were transiently transfected with pcDNA 3.1(+) vector (Invitrogen) encoding RLuc, RLuc8, RLuc8.6–535, RLuc8.6-535SG, RLuc8.6-547, ALuc16, ALuc23, or NanoLuc, using a lipofection reagent (TransIT-LT1, Mirus). The cells were incubated two days in a CO_2_ incubator (Sanyo). The cells were trypsinized and subcultured into a 96-well black-frame optical-bottom microplate (Thermo Fisher Scientific). The cells were further incubated in the CO_2_ incubator until reach 90% of confluence. The cells were then lysed with a lysis buffer (Promega) for 20 min and an aliquot of the lysates (40 µL) were transferred into a PCR tube (200 µL volume). The consequent BL spectra were determined after injection of each luciferin dissolved in 40 µL HEPES buffer (50 mM, pH 7.2, Thermo Fisher) using a precision spectrophotometer (AB-1850, ATTO) simultaneously acquiring entire wavelengths of BL. The light integration time was 1, 2, or 5 min.

The cellular concentrations of the luciferases are unclear because the expression levels are not controllable. Instead, we conducted all the studies hereafter with equal amounts of the substrates and the fixed number of the cells (10,000 per well on a 96well microplate).

### Determination of the luciferase specificity of the CTZ variants

The MDA-MB-231 cells were raised in a 6-well microplate (Nunc) until reach 70% of confluence. The cells were transiently transfected with pcDNA3.1(+) vector encoding GLuc, MLuc, NanoLuc, RLuc8.6-535, RLuc8.6-535SG, ALuc16, or ALuc23. The cells were further incubated in a CO_2_ incubator for 2 days. The cells were then subcultured into a 96-well black-frame optical-bottom microplate and incubated one more day in the CO_2_ incubator.

The culture media of the microplates are decanted and washed once with a phosphate-buffered saline (PBS). The wells in the microplate were randomly separated into two sections. The following live cell imaging was conducted through simultaneously injecting a series of CTZ analogues dissolved in 40 µL HEPES buffer into the wells in a section using a 12-channel micropipette. The consequent BL intensities were determined with an IVIS imaging system (PerkinElmer). On the other hand, the wells in the other section were lysed with a lysis buffer (Promega) for 20 min. 20 µL of the lysates were moved to a fresh 96-well black-frame optical-bottom microplate. The lysates in the microplate was simultaneously mixed with 40 µL of CTZ analogues dissolved in HEPES buffer using a 12-channel micropipette. The optical intensities were immediately determined with the IVIS imaging system (PerkinElmer).

### Determination of the kinetic parameters of the BL according to the colenterazine (CTZ) analogues Lineweaver–Burk

The kinetic parameters of the BL according to the coelenterazine (CTZ) analogues were determined with Lineweaver**–**Burk equation (Table 1).

A series of reaction solutions were prepared beforehand. Firstly, the CTZ variants were dissolved in HEPES buffer (50 mM, pH 7.2) to be 0.02–100 µM, and deployed in a 96-well black-frame optical-bottom microplate. Secondly, the recombinant marine luciferases (RLuc8.6-535 and ALuc16) were also dissolved in the same HEPES buffer to be 0.2 µM, and primed in the automatic injectors of a microplate reader (TriStar2 S LB942, Berthold), respectively. The substrates were placed in the microplate (final concentrations: 0.01–50 µM) beforehand and the microplate was then set on the sample stage of the microplate reader. The marine luciferase (final concentration: 0.1 µM) primed in the reader injector were injected into each well of the microplate and the corresponding BL intensities were recorded every 0.1 s during the initial five seconds. The final concentrations of the substrates were at 0.1, 0.25, 0.5, 1, 2.5, 5, 10, 25, 50, and 100 µM. The measurements were quadruplicated for the following statistical analyses (n = 4). The *K*_m_ and *V*_max_ values were calculated from Lineweaver–Burk plots using the Enzyme Kinetics Wizard in the commercially available SigmaPlot 13.0 software package (Systat Software Inc., San Jose, CA).

### Synthesis of CTZ analogues 1a–d and 2a–d

The CTZ analogues **1a–d** and **2a–d** were synthesized according to the following scheme (Figs. S7 and S8): the starting chemicals, aldehyde **5a–c**, were boron-Wittig reacted with [(pinacolato) boryl] methane to produce pinacol borane esters **6a–c**. Separately, 2-amino-3-benzyl-5-Bromoaminopyrazine **4a** was synthesized by a coupling reaction of commercially available 2-amino-3,5-dibromoaminopyrazine **3** with benzylmagnesium chloride and bis (triphenylphosphine) palladium (II) dichloride. We further conducted a Suzuki–Miyaura coupling reaction using **3** and a phenylboronic acid to produce 2-amino-3-phenyl-5-bromoaminopyrazine (**4b**). The made **4a** and **4b** were additionally reacted with 4-(dimethylamino) phenylboronic acid or **6a-c** through Suzuki–Miyaura coupling to create the aminopyrazine derivatives **7a–d** and **8a–d**. Finally, the synthesized aminopyrazine derivatives **7a–d** and **8a–d** were reacted with the ketoacetal derivatives **9** and **10**, respectively, and condensed and cyclized under hydrochloric acid conditions. The synthesized CTZ analogues were named **1a–d** and **2a–d**, respectively.

### Synthesis of CTZ analogues 3a–d

The CTZ analogues **3a–d** were synthesized according to the following scheme (Fig. S9): An aminopyrazine derivative **12a** was firstly synthesized by a Stille coupling reaction between **4b** and 2-(tributyltin)pyridine (**11a**). The aminopyrazine derivatives **12b–d** were separately synthesized by Suzuki–Miyaura coupling with **4b** and boronic acids **11b–d**. Finally, the obtained aminopyrazine derivatives **12a-d** were reacted with a ketoacetal derivative **10**, and further condensed and cyclized under hydrochloric acid conditions. The consequent CTZ analogues were named **3a–d**.

### Synthetic procedures of the CTZ analogues general

The starting materials, reagents, and solvents were purchased and used without further purification. We used Silica gel 70 F254 TLC plates (Wako) for analytical Thin-layer chromatography (TLC), while Silica gel 60 N (spherical, neutral, Kanto Chemical) were for column chromatography. For preparative flash chromatography, used were an automated system (Smart Flash EPCLC AI-580S, Yamazen Corp., Japan) equipped with universal columns of silica gel. Melting points and IR spectra were determined with a MP-500P (Yanaco) and a Nicolet 6700 spectrometer with anATR attachment, respectively. ^1^H and ^13^C NMR spectra were determined on a JEOL ECA-500 instrument (500 MHz for ^1^H and 126 MHz for ^13^C). Mass spectra were obtained with a high-resolution electro-spray ionization mass spectrometer, JMS-T100LC (JEOL). UV/visible absorption spectra were determined with a spectrophotometer, Cary 60 (Agilent Technologies) (scan speed 600 nm/min; data interval 1 nm). BL and chemiluminescence spectra were measured with a precision spectrophotometer, AB-1850 (ATTO) (data interval: 1 nm). BL intensities were monitored using luminometers, AB-2270 (ATTO) and GL-201A (Microtec Co.). BL imaging was performed with a multifunctional in vivo imaging system (IVIS Spectrum, PerkinElmer).

### DFT and TD DFT calculations of oxy-1a′–d′, oxy-2a′–d′, oxy-1a′–d′ (an), and oxy-2a′–d′ (an)

Density functional theory (DFT) calculations were performed with the Gaussian 09 program (Rev. D.01)^32^. DFT includes the B3LYP function with the 6-31 + G(d) basis set^22–24^ and IEF-PCM approximation in DMSO^27^. The molecular structures of oxy-**1a**′–**d**′, oxy-**2a**′–**d**′, oxy-**1a**′–**d**′ (an), and oxy-**2a**′–**d**′ (an) were optimized by DFT calculations as shown in Fig. S10. Based on their optimized structures, time-dependent (TD) DFT calculations were carried out to give the properties of the electronic transitions including the S_0_ → S_1_ transitions. Molecular graphics were made with GaussView, Version 5^33^.

## Supplementary information

Supplementary Information
