# Supplementary material for: Color-tunable bioluminescence imaging portfolio for cell imaging
Source: Sci Rep. 2021 Jan 26;11:2219. doi: 10.1038/s41598-021-81430-1 (PMC7838199; doi:10.1038/s41598-021-81430-1)
Supplement: Supplementary file 1 — Supplementary Information [file 41598_2021_81430_MOESM1_ESM.docx]

Suppl. Information

Color-Tunable Bioluminescence Imaging Portfolio for Cell Imaging

**Shota Tamaki,^[a]^** ^‡^**Nobuo Kitada,^[a]^** ^‡^ **Masahiro Kiyama,^[a]^ Rika Fujii,^[b]^ Takashi Hirano,^[a]^ Sung Bae Kim,*^[b]^ Shojiro Maki*^[a]^**

[a] Mr. S. Tamaki, Dr. N. Kitada, Dr. M. Kiyama, Prof. Dr. T. Hirano, Prof. Dr. S. Maki
Department of Engineering Science, Graduate School of Informatics and Engineering,
The University of Electro-Communications
1-5-1 Chofugaoka, Chofu, Tokyo, 182-8585, Japan
E-mail: s-maki@uec.ac.jp

[b] Dr. R. Fujii, Dr. S. B. Kim
Research Institute for Environmental Management Technology
National Institute of Advanced Industrial Science and Technology (AIST)
16-1 Onogawa, Tsukuba, Ibaraki 305-8569, Japan
E-mail: kimu-sb@aist.go.jp

‡ Equally Contributing Authors

**Contents**

1. Experimental Procedures Page 2
2. BL spectra of coelenterazine (CTZ) variants according to marine luciferases Page 5
3. Synthesis of CTZ analogues Page 10
4. Computational methods Page 26
5. **Experimental procedures**
   1. **Preparation of the plasmids encoding a marine luciferase.**

The pcDNA 3.1(+) plasmids (Invitrogen) encoding various marine luciferases were obtained from our previous studies ^1 2 3 4^: The luciferases include *Renilla* luciferase (RLuc), *Renilla* luciferase 8 (RLuc8), *Renilla* luciferase 8.6-535 (RLuc8.6-535), *Renilla* luciferase 8.6-535SG (RLuc8.6-535SG), *Renilla* luciferase 8.6-547 (RLuc8.6-547), *Gaussia* luciferase (GLuc, GenBank AAG54095.1), Metridia longa luciferase (MLuc), NanoLuc, Artificial Luciferase 16 (ALuc16, GenBank MF817967), and Artificial Luciferase 23 (ALuc23, MF817968).

Briefly, A pMetluc2 vector encoding Metridia longa luciferase (MLuc) was purchased from Clontech (Mountain View, CA). The RLuc variants (RLuc8, RLuc8.6-535, and RLuc8.6-535SG) were generously gifted by Dr. Sanjiv S. Gambhir (Stanford University) or Dr. Moritoshi Sato (Tokyo University). The cDNAs encoding the other marine luciferases were custom-synthesized by Eurofins Genomics, based on the open information of the cDNA sequences in the National Center for Biotechnology Information (NCBI) database.

The cDNA fragments were ligated and subcloned in pcDNA3.1(+) vectors for mammalian cell expression. The overall sequence fidelity was confirmed with a sequencing service provided by Eurofins Genomics (Tokyo).

- 1. **Determination of bioluminescence (BL) spectra of the CTZ analogues.**

The BL spectra of the CTZ analogues were determined according to various luciferases (Figure 2 and Figures S1, S2, and S3).

The MDA-MB-231 cells derived from epithelial, human breast cancer cell line were originally cultured in 6-well microplates (Nunc) using a Dulbecco's Modified Eagle Medium (DMEM) supplemented with 10% fetal bovine serum (FBS) and 1% penicillin-streptomycin (final concentration: 100 U/mL). The cells were transiently transfected with pcDNA 3.1(+) vector (Invitrogen) encoding RLuc, RLuc8, RLuc8.6-535, RLuc8.6-535SG, RLuc8.6-547, ALuc16, ALuc23, or NanoLuc, using a lipofection reagent (TransIT-LT1, Mirus). The cells were incubated two days in a CO_2_ incubator (Sanyo). The cells were trypsinized and subcultured into a 96-well black-frame optical-bottom microplate (Thermo Fisher Scientific). The cells were further incubated in the CO_2_ incubator until reach 90% of confluence. The cells were then lysed with a lysis buffer (Promega) for 20 minutes and an aliquot of the lysates (40 μL) were transferred into a PCR tube (200 μL volume). The consequent BL spectra were determined after injection of each luciferin dissolved in 40 μL HEPES buffer (50 mM, pH 7.2, Thermo Fisher) using a precision spectrophotometer (AB-1850, ATTO) simultaneously acquiring entire wavelengths of BL. The light integration time was 1, 2, or 5 minutes.

- 1. **Determination of the luciferase specificity of the CTZ variants.**

The MDA-MB-231 cells were raised in a 6-well microplate (Nunc) until reach 70% of confluence. The cells were transiently transfected with pcDNA3.1(+) vector encoding GLuc, MLuc, NanoLuc, RLuc8.6-535, RLuc8.6-535SG, ALuc16, or ALuc23. The cells were further incubated in a CO_2_ incubator for two days. The cells were then subcultured into a 96-well black-frame optical-bottom microplate and incubated one more day in the CO_2_ incubator.

The culture media of the microplates are decanted and washed once with a phosphate-buffered saline (PBS). The wells in the microplate were randomly separated into two sections. For live cell imaging studies, the wells in a section was simultaneously injected with a series of CTZ analogues dissolved in 40 μL HEPES buffer using a 12-channel micropipette. The consequent BL intensities were determined with an IVIS imaging system (PerkinElmer). On the other hand, the wells in the other section were lysed with a lysis buffer (Promega) for 20 minutes. An aliquot of the lysates (20 μL) were transferred to a fresh 96-well black-frame optical-bottom microplate. The lysates in the microplate was simultaneously mixed with 40 μL of CTZ analogues dissolved in HEPES buffer using a 12-channel micropipette. The optical intensities were immediately determined with the IVIS imaging system (PerkinElmer).

- 1. **Determination of the kinetic parameters of the BL according to the colenterazine (CTZ) analogues**

**Lineweaver–Burk**

The kenetic parameters of the BL according to the coelenterazine (CTZ) analogues were determined with Lineweaver**–**Burk equation (Table 1).

A series of reaction solutions were prepared beforehand. Firstly, the CTZ variants were dissolved in HEPES buffer (50 mM, pH 7.2) to be 0.02 - 100 μM, and deployed in a 96-well black-frame optical-bottom microplate. Secondly, the recombinant marine luciferases (RLuc8.6-535 and ALuc16) were also dissolved in the same HEPES buffer to be 0.2 μM, and primed in the automatic injectors of a microplate reader (TriStar2 S LB942, Berthold), respectively. The prepared substrates in the microplate (final concentrations: 0.01 - 50 μM) was then set in the microplate reader, the wells of which were injected with the primed marine luciferase (final concentration: 0.1 μM) and the corresponding BL intensities were recorded every 0.1 second. The final concentrations of the substrates ranged from 0.01 to 50 μM. The measurements were quadruplicated for the following statistic analyses (n=4). The *K*_m_ and *V*_max_ values were calculated from Lineweaver–Burk plots using the Enzyme Kinetics Wizard in the commercially available SigmaPlot 13.0 software package (Systat Software Inc., San Jose, CA).

1.
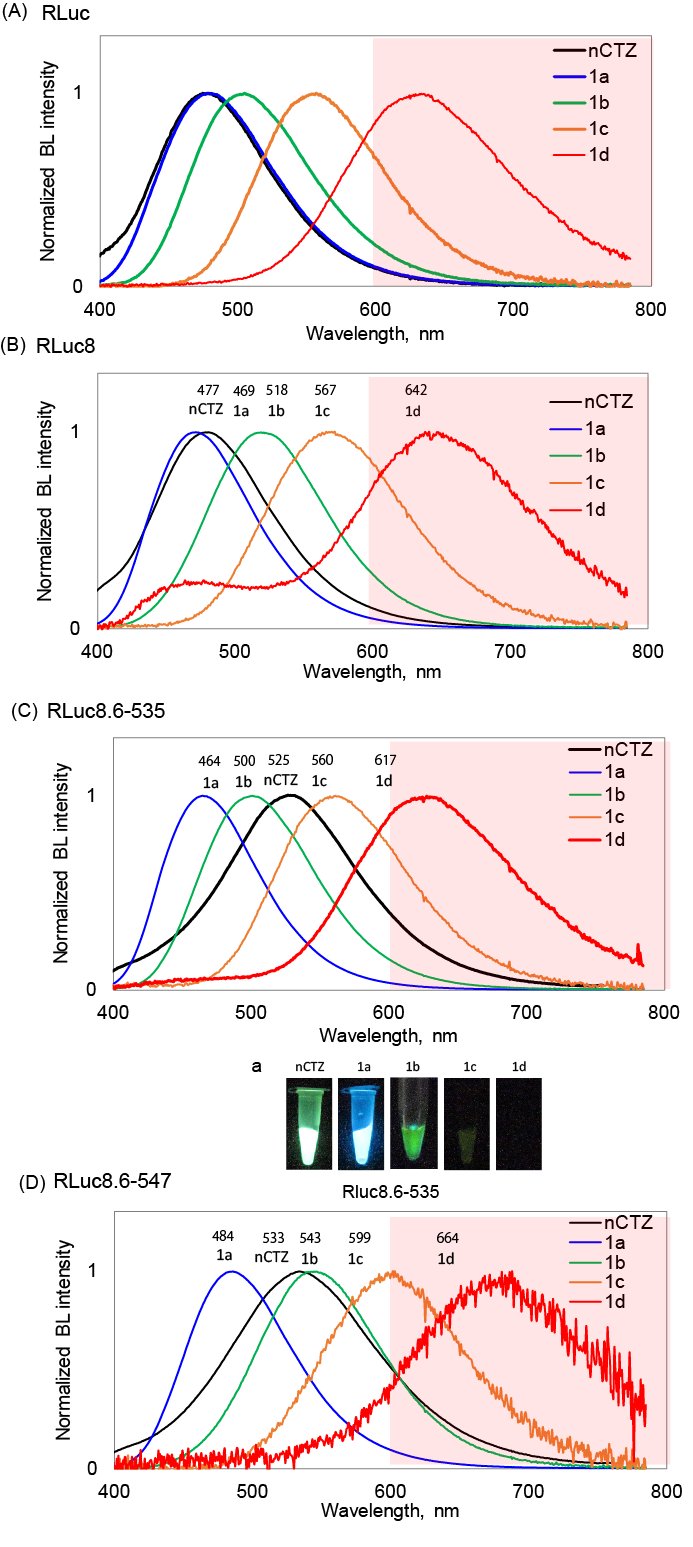
**BL spectra of CTZ variants according to marine luciferases**

**Figure S1**. Characterization of the BL-spectral properties of 1-series CTZ analogues with RLuc derivatives. (A) The BL spectra of 1-series CTZ analogues with RLuc. (B) The BL spectra of 1-series CTZ analogues with RLuc8. (C) The BL spectra of 1-series CTZ analogues with RLuc8.6-535. (D) The BL spectra of 1-series CTZ analogues with RLuc8.6-547.


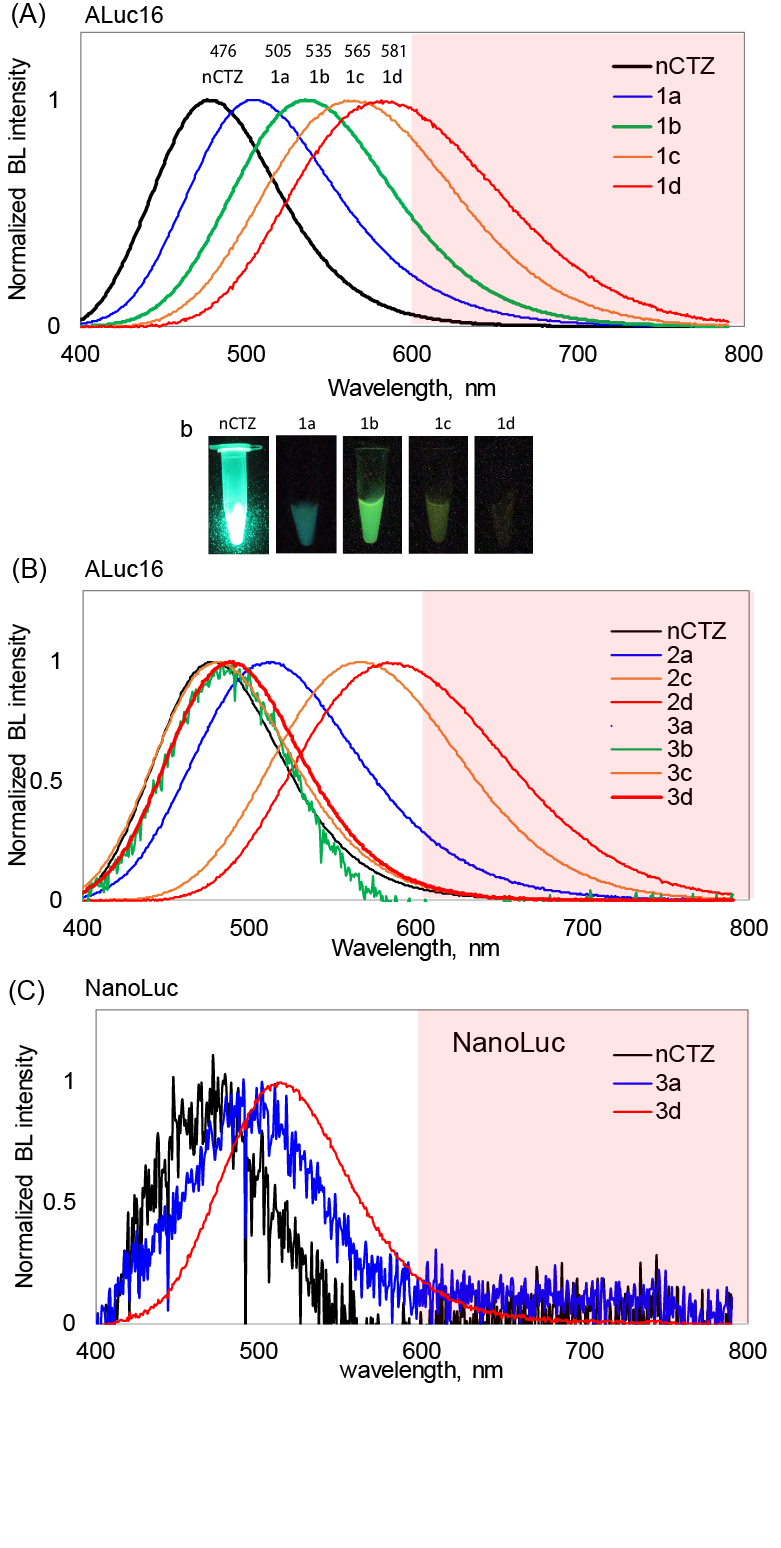


**Figure S2**. Characterization of the BL spectra of the new CTZ analogues with ALuc16 and NanoLuc. (A) The BL spectra of 1-series CTZ analogues with ALuc16. (B) The BL spectra of 2- and 3-series CTZ analogues with ALuc16. (C) The BL spectra of 3-series CTZ analogues with NanoLuc.


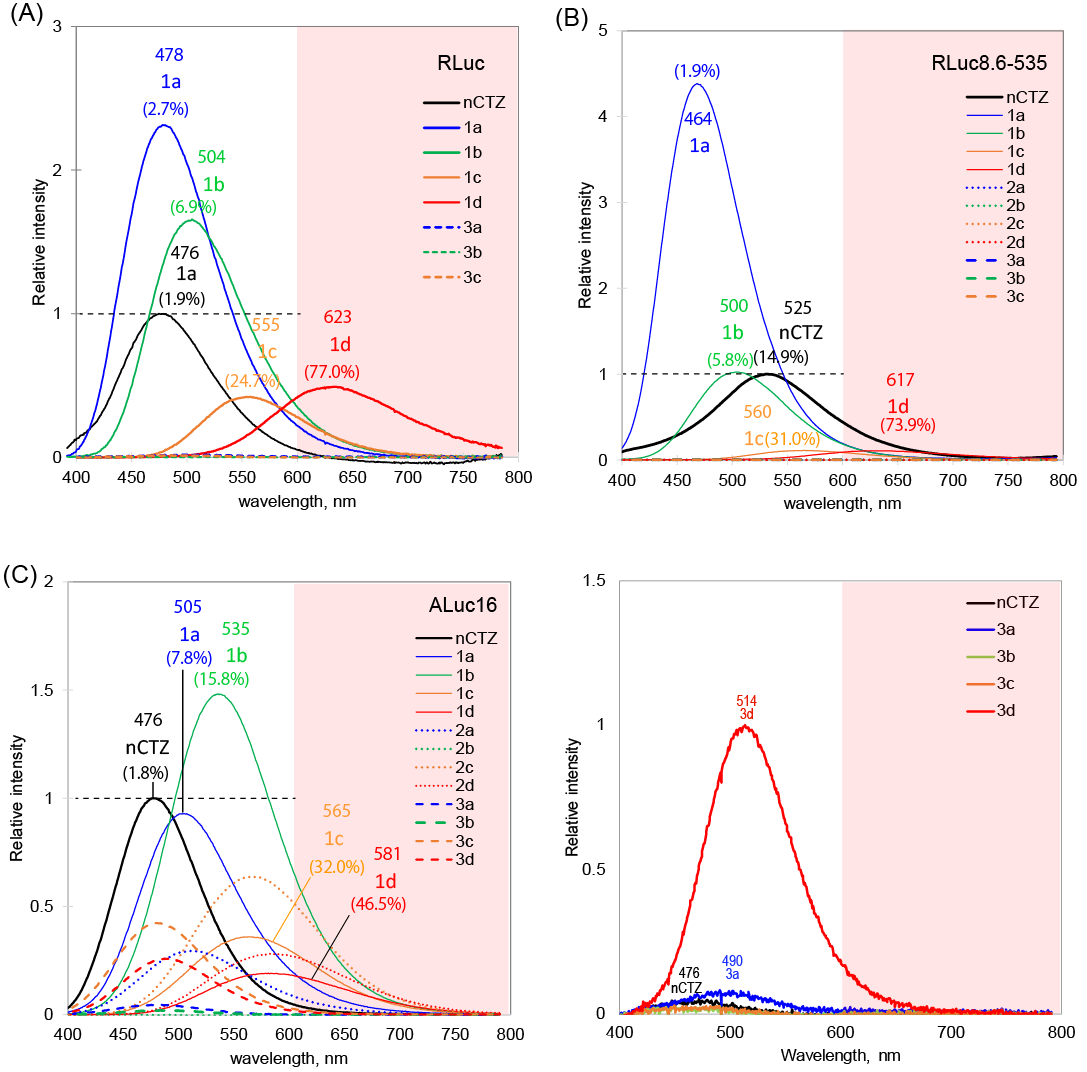


**Figure S3**. Characterization of the relative BL spectra of the made CTZ analogues, compared to CTZ. (A) The BL spectra of 1- and 3-series CTZ analogues with RLuc, which was normalized to the BL spectra of CTZ with RLuc. (B) The BL spectra of the made CTZ analogues with RLuc8.6-535, which was normalized to the BL spectra of CTZ with RLuc8.6-535. (C) The BL spectra of the made CTZ analogues with ALuc16, which was normalized to the BL spectra of CTZ with ALuc16. (D) The BL spectra of the 3-series CTZ analogues with NanoLuc, which was normalized to the BL spectra of **3d** with NanoLuc.


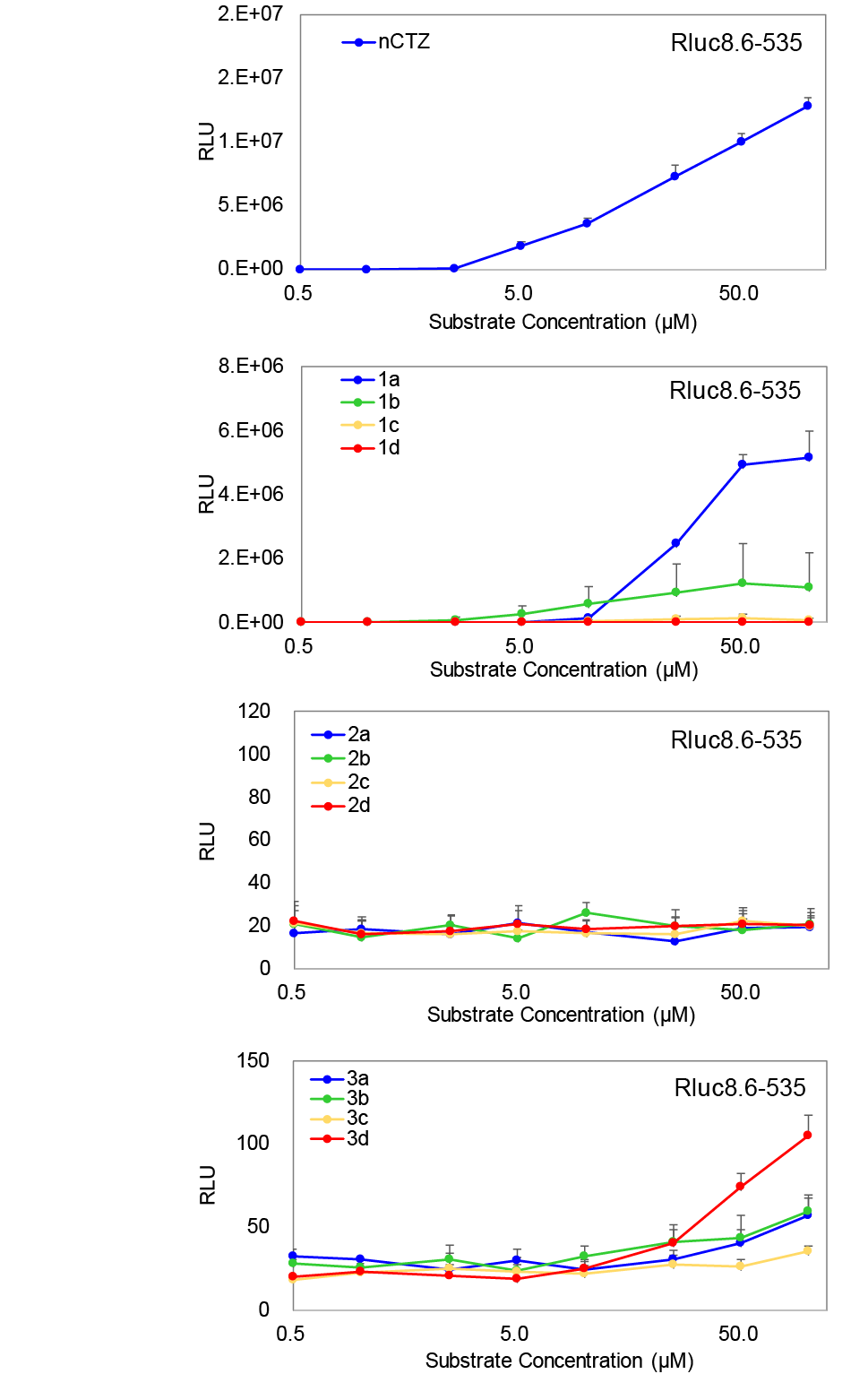


**Figure S4**. Characterization of the BL intensities of RLuc8.6-535 with varying concentrations of the made CTZ analogues. (A) The BL intensities of RLuc8.6-535 according to varying concentrations of CTZ. (B) The BL intensities of RLuc8.6-535 according to varying concentrations of 1-sereis CTZ analogues. (C) The BL intensities of RLuc8.6-535 according to varying concentrations of 2-series CTZ analogues. The BL intensities of RLuc8.6-535 according to varying concentrations of 3-series CTZ analogues.


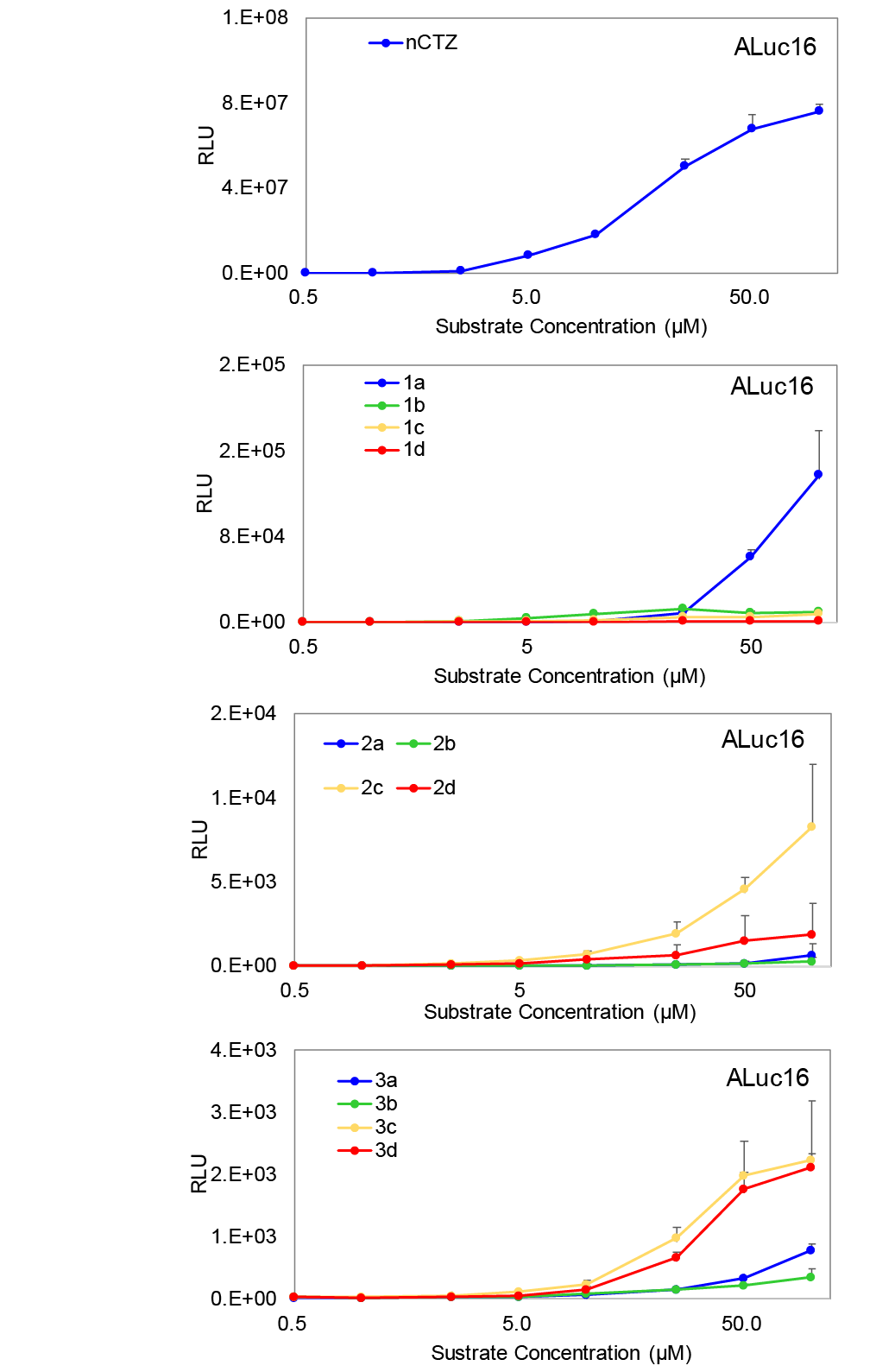


**Figure S5**.

Characterization of the BL intensities of ALuc16 with varying concentrations of the made CTZ analogues. (A) The BL intensities of ALuc16 according to varying concentrations of CTZ. (B) The BL intensities of ALuc16 according to varying concentrations of 1-series CTZ analogues. (C) The BL intensities of ALuc16 according to varying concentrations of 2-series CTZ analogues. (D) The BL intensities of ALuc16 according to varying concentrations of 3-series CTZ analogues.

**Figure S6.** Time course of the BL intensities of RLuc8.6-535 (A) and ALuc16 (B) according to substrates.

1. **Synthesis of CTZ Analogues**

**3.1 Scheme of the organic synthesis of the CTZ analogues**

The novel CTZ analogues specified in Figure S7 were synthesized according to the following schemes.

**Synthesis of CTZ analogues 1a-d and 2a-d** The CTZ analogues **1a-d** and **2a-d** were synthesized according to the following scheme (**Figure S8**): the starting chemicals, aldehyde **5a-c**, were boron-Wittig reacted with [(pinacolato) boryl] methane to produce pinacol borane esters **6a-c**. Separately, 2-amino-3-benzyl-5-Bromoaminopyrazine **4a** was synthesized by a coupling reaction of commercially available 2-amino-3,5-dibromoaminopyrazine **3** with benzylmagnesium chloride and bis (triphenylphosphine) palladium (II) dichloride. We further conducted a Suzuki-Miyaura coupling reaction using **3** and a phenylboronic acid to produce 2-amino-3-phenyl-5-bromoaminopyrazine (**4b**). The made **4a** and **4b** were additionally reacted with 4-(dimethylamino) phenylboronic acid or **6a-c** through Suzuki-Miyaura coupling to create the aminopyrazine derivatives **7a-d** and **8a-d**. Finally, the synthesized aminopyrazine derivatives **7a-d** and **8a-d** were reacted with the ketoacetal derivatives **9** and **10**, respectively, and condensed and cyclized under hydrochloric acid conditions. The synthesized CTZ analogues were named **1a-d** and **2a-d**, respectively.

**Synthesis of CTZ analogues 3a-d** The CTZ analogues **3a-d** were synthesized according to the following scheme (**Figure S9**): An aminopyrazine derivative **12a** was firstly synthesized by a Stille coupling reaction between **4b** and 2-(tributyltin)pyridine (**11a**). The aminopyrazine derivatives **12b-d** were separately synthesized by Suzuki-Miyaura coupling with **4b** and boronic acids **11b-d**. Finally, the obtained aminopyrazine derivatives **12a-d** were reacted with a ketoacetal derivative **10**, and further condensed and cyclized under hydrochloric acid conditions. The consequent CTZ analogues were named **3a-d**.

**
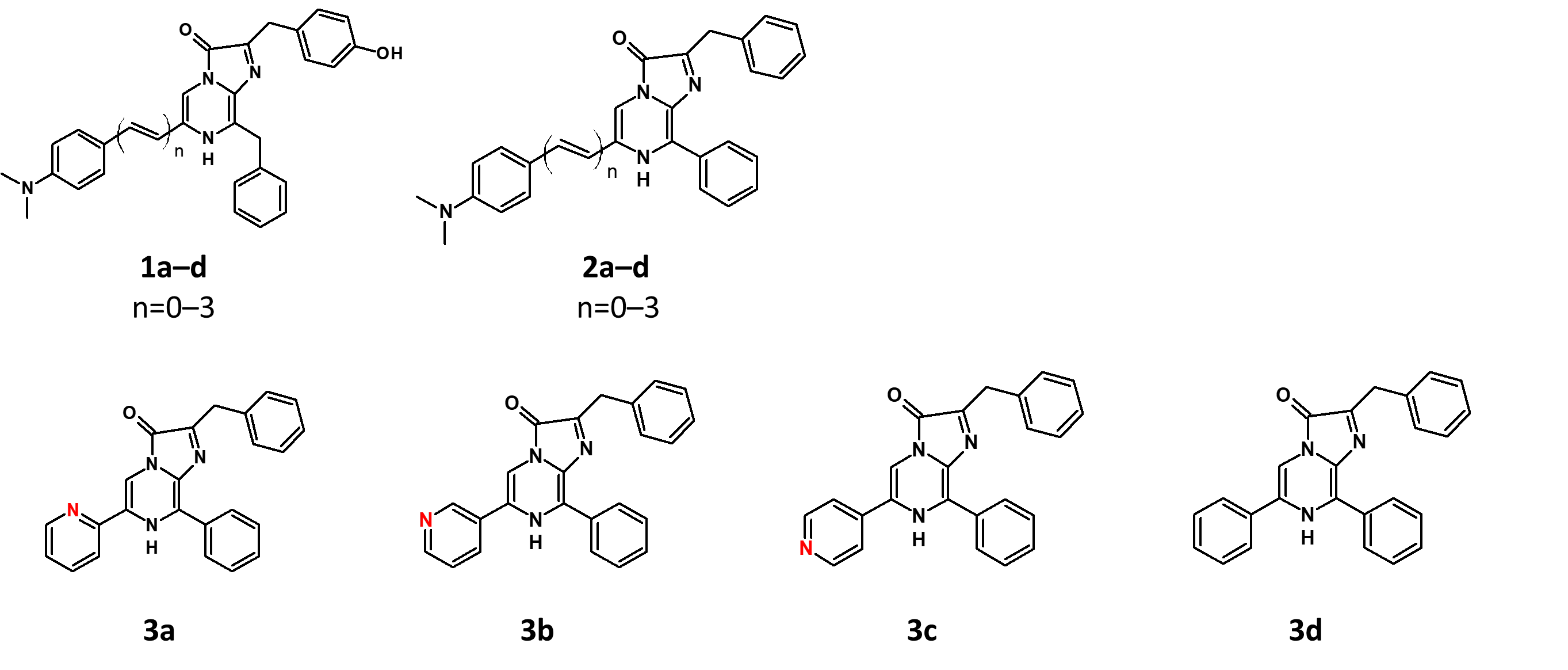
**

**Figure S7.** The structures of **1a-d**, **2a-d** **3a-d**

- 1. **Synthetic procedures of the CTZ analogues**

**General**

　Commercially available reagents and solvents were used without further purification. Wako Silica gel 70 F254 TLC plates were used for analytical TLC, and Kanto Chemical Silica gel 60 N (spherical, neutral) were used for column chromatography. For preparative flash chromatography, an automated system (Smart Flash EPCLC AI-580S, Yamazen Corp., Japan) were used with universal columns of silica gel. Melting points were measured with a Yanaco MP-500P.IR spectra were obtained with a Nicolet 6700 spectrometer with anATR attachment.1H and 13C NMR spectra were recorded on a JEOL ECA-500 instrument (500 MHz for 1H and 126 MHz for 13C). High-resolution electro-spray ionization mass spectra were obtained with a JEOL JMS-T100LC mass spectrometer. UV/visible absorption spectra were obtained using an Agilent Technologies Cary 60 spectrophotometer (scan speed 600 nm/min; data interval 1 nm). Bioluminescence and chemiluminescence spectra were measured with an ATTO AB-1850 spectrophotometer (data interval 1 nm). Bioluminescence intensities were monitored using an ATTO AB-2270 and a Microtec Co. GL-201A luminometer. BLI was performed with a multifunctional *in vivo* imaging system (IVIS Spectrum, PerkinElmer).


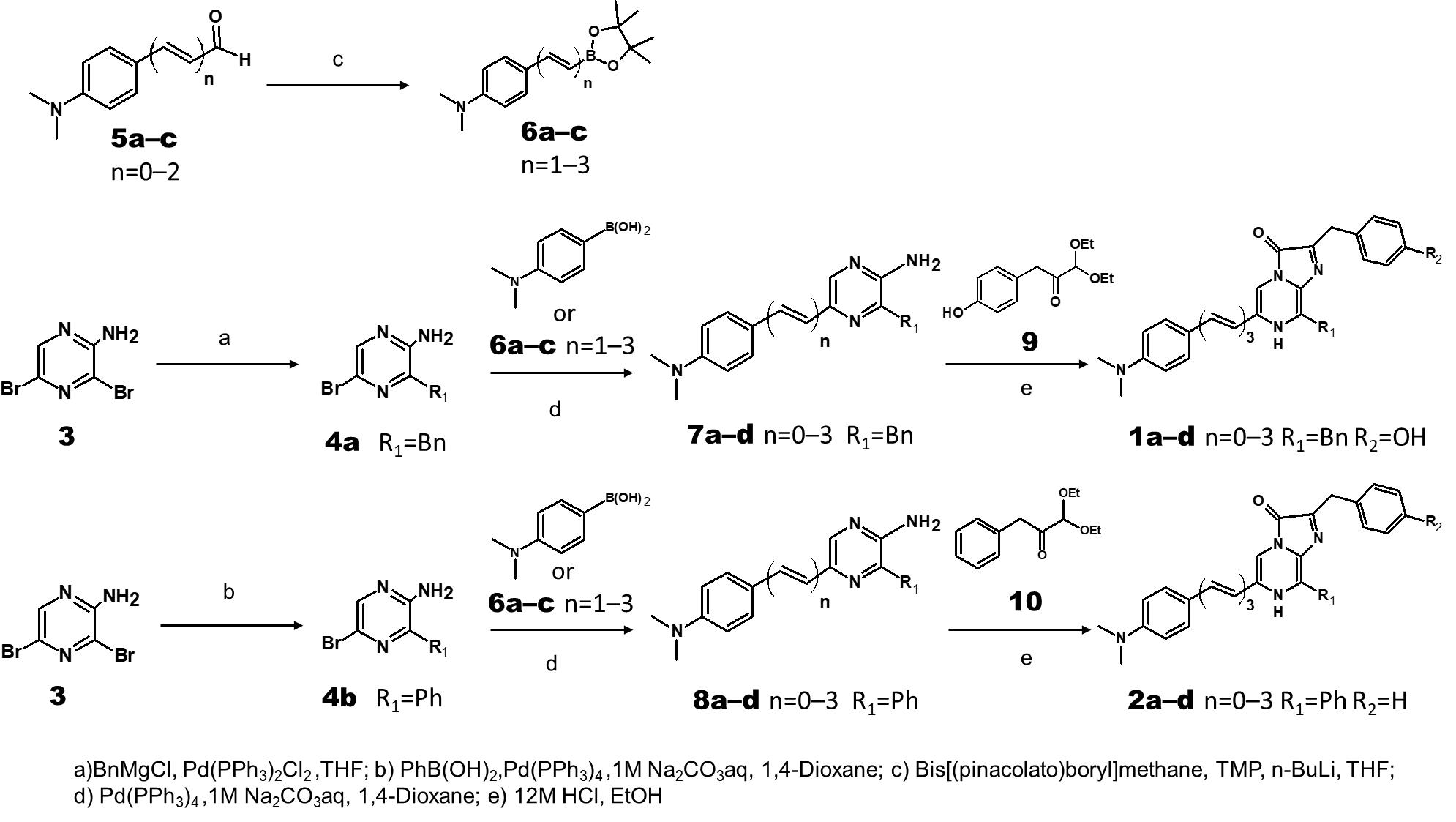


**Figure S8.** Synthetic scheme for **1a-d** and **2a-d**

(Compounds **3**, **4a**, **4b**, **9**, **10** are known procedures)

**(*E*)-N,N-dimethyl-4-(2-(4,4,5,5-tetramethyl-1,3,2-dioxaborolan-2-yl)vinyl)aniline (6a)**

A 1.6 M n-butyllithium hexane solution (3.0 mmol) was added to a solution of 2,2,6,6-tetramethylpiperidine (3.0 mmol) in tetrahydrofuran (1 mL) at 0 ° C under an argon atmosphere, followed by stirring for 5 minutes. A solution of bis ([pinacolato] boryl) methane (3.0 mmol) in tetrahydrofuran (1 mL) was added to the reaction mixture, and the mixture was stirred for 5 minutes. Subsequently, the reaction mixture was cooled to −78 ° C., a solution of 4-dimethylaminobenzaldehyde (2.0 mmol) in tetrahydrofuran (1 mL) was slowly added, and the mixture was stirred for 4 hours. Thereafter, the temperature was raised to room temperature, toluene was added, and the mixture was concentrated under reduced pressure. The obtained residue was separated by flash chromatography (hexane / ethyl acetate = 4/1) to give **6a** (268 mg, 0.98 mmol, 49%) as yellow crystals.

; IR (neat, *ν*, cm^-1^): 1600, 1351, 1319, 1136, 1001

; ^1^H-NMR (500 MHz, CDCl_3_) *δ* 7.36 (d, *J* = 9.2 Hz, 2 H), 7.34 (d, *J* = 17.8 Hz, 1 H), 6.61 (d, *J* = 9.2 Hz, 2 H), 5.91 (d, *J* = 18.3 Hz, 1 H), 2.91 (s, 6 H), 1.28 (s, 12 H)

; ^13^C-NMR (125 MHz, CHLOROFORM-D) *δ* 150.65, 149.60, 128.10, 125.55, 111.64, 82.67, 39.94, 24.57

; HR-ESI-MS: *m/z*: [M+H]^+^ calcd for C_16_H_24_BNO_2_, 274.1978; found, 274.1991.

Di and triene-pinacol borane esters **6b** and **6c** were prepared with a similar procedure to the preparation of **6a**.

**N,N-dimethyl-4-((1*E*,3*E*)-4-(4,4,5,5-tetramethyl-1,3,2-dioxaborolan-2-yl)buta-1,3-dien-1-yl)aniline (6b)**

(yield 37%), yellow crystals:

; mp. 98–102 °C

; IR (neat, *ν*, cm^-1^): 1594, 1299, 1134, 1012

; ^1^H-NMR (500 MHz, CDCl_3_) *δ* 7.30 (d, *J* = 8.6 Hz, 2 H), 7.16 (dd, *J* = 9.0 Hz, 1 H), 6.60–6.67 (comp., 4 H), 5.54 (d, *J* = 17.8 Hz, 1 H), 5.25 (s, 1 H), 2.94 (s, 6 H), 1.27 (s, 12 H)

; ^13^C-NMR (125 MHz, CDCl_3_) *δ* 150.74, 150.34, 136.68, 128.00, 126.29, 124.87, 112.09, 82.90, 40.21, 24.69

; HR-ESI-MS: *m/z*: [M+H]^+^ calcd for C_18_H_26_BNO_2_, 300.2135; found, 300.2139.

**N,N-dimethyl-4-((1*E*,3*E*,5*E*)-6-(4,4,5,5-tetramethyl-1,3,2-dioxaborolan-2-yl)hexa-1,3,5-trien-1-yl)aniline (6c)**

(yield 21%), yellow crystals:

; IR (neat, *ν*, cm^-1^): 1590, 1574, 1362, 1332, 1294, 1261, 1147, 1129, 1102, 1015, 972

; ^1^H-NMR (500 MHz, CDCl_3_) *δ* 7.31 (d, *J* = 9.2 Hz, 2 H), 7.09 (dd, *J* = 17.5, 10.6 Hz, 1 H), 6.74–6.44 (comp., 5 H), 6.34 (dd, *J* = 14.9, 10.9 Hz, 1 H), 5.53 (d, *J* = 17.2 Hz, 1 H), 2.98 (s, 6 H), 1.28 (s, 12 H)

; ^13^C-NMR (125 MHz, CDCl_3_) *δ* 150.03, 150.01, 137.61, 135.16, 132.14, 127.64, 125.12, 124.16, 112.06, 82.88, 40.14, 24.63

; HR-ESI-MS: *m/z*: [M+H]^+^ calcd for C_20_H_28_BNO_2_, 326.2291; found, 326.2297.

**3-benzyl-5-(4-(dimethylamino)phenyl)pyrazin-2-amine** (**7a**)

2-amino-3-benzyl-5-bromopyrazine **4a** (0.3 mmol), 4-(dimethylamino) phenylboronic acid (0.45 mmol), tetrakis (triphenylphosphine) palladium (0) (0.0015 mmol) in 1,4 -Dissolved in dioxane (1 mL), added 1M aqueous sodium carbonate solution (1 mL) under an argon atmosphere, and stirred at 90 ° C for 3 hours. After completion of the reaction, the reaction mixture was extracted with ethyl acetate (30 mL × 2), and washed with saturated saline. The organic layers were combined, dried over sodium sulfate, and then concentrated under reduced pressure. The obtained residue was separated by flash chromatography (hexane / ethyl acetate = 1/1) to give 2-amino-3-benzyl-5-dimethylaminophenylpyrazine **7a** (73 mg, 0.24 mmol, 80%). Obtained as yellow crystals.

; mp. 159–160 ° C; IR (neat, *ν*, cm^-1^)

: 1607, 1463, 1439, 1424, 1222, 1122, 1062

; ^1^H-NMR (500 MHz, CDCl_3_) *δ* 8.31 (s, 1 H), 7.84 (d, *J* = 8.6 Hz, 2 H), 7.37–7.19 (comp., 5 H), 6.80 (d, *J* = 9.2 Hz, 2 H), 4.30 (s, 2 H), 4.16 (s, 2 H), 3.00 (s, 6 H)

; HR-ESI-MS: *m/z*: [M+H]^+^ calcd for C_19_H_21_N_4_, 305.1766; found, 305.1770.

Aminopyrazine derivative **7b-d** were synthesized from the pinacol borane ester **6a-c** in the similar procedure to the preparation of **7a**.

**(*E*)-3-benzyl-5-(4-(dimethylamino)styryl)pyrazin-2-amine (7b)**

(yield 84%), yellow crystals:

; IR (neat, *ν*, cm^-1^): 1605, 1522, 1452, 1396, 1360, 1184, 1131, 959

; ^1^H-NMR (500 MHz, CDCl_3_) *δ* 8.00 (s, 1 H), 7.47–7.41 (comp., 3 H), 7.34–7.24 (comp., 5 H), 6.92 (d, *J* = 15.5 Hz, 1 H), 6.72 (d, *J* = 8.6 Hz, 2 H), 4.30 (s, 2 H), 4.16 (s, 2 H), 3.00 (s, 6 H)

; HR-ESI-MS: *m/z*: [M+H]^+^ calcd for C_21_H_23_N_4_, 331.1923; found, 331.1927.

**3-benzyl-5-((1*E*,3*E*)-4-(4-(dimethylamino)phenyl)buta-1,3-dien-1-yl)pyrazin-2-amine (7c)**

(yield 85%), yellow crystals:

; IR (neat, *ν*, cm^-1^): 1594, 1517, 1451, 1402, 1349, 1156, 990

; ^1^H-NMR (500 MHz, CDCl_3_) *δ* 7.90 (s, 1 H), 7.35–7.23 (comp., 8 H), 6.80 (dd, *J* = 15.3, 11.0 Hz, 1 H), 6.69–6.65 (comp., 3 H), 6.55 (d, *J* = 15.5 Hz, 1 H), 4.40 (s, 2 H), 4.11 (s, 2 H), 2.96 (s, 6 H)

; ^13^C-NMR (125 MHz, CDCl_3_) *δ* 151.07, 150.07, 141.88, 140.89, 138.88, 136.68, 134.20, 131.14, 128.93, 128.48, 127.58, 126.97, 126.21, 125.72, 124.75, 112.33, 41.26, 40.35

; HR-ESI-MS: *m/z*: [M+H]^+^ calcd for C_23_H_25_N_4_, 357.2079; found, 357.2073.

**3-benzyl-5-((1*E*,3*E*,5*E*)-6-(4-(dimethylamino)phenyl)hexa-1,3,5-trien-1-yl)pyrazin-2-amine (7d)** (yield 89%), yellow crystals:

; IR (neat, *ν*, cm^-1^): 1582, 1516, 1451, 1402, 1347, 995

; ^1^H-NMR (500 MHz, CDCl_3_) *δ* 7.90 (s, 1 H), 7.33–7.22 (comp., 8 H), 6.73 (dd, 15.5, 10.9 Hz, 1 H), 6.67 (d, *J* = 8.6 Hz, 2 H), 6.61–6.41 (m, 4 H), 4.48–4.35 (2 H), 4.17–4.06 (2 H), 2.96 (s, 6 H)

; ^13^C-NMR (125 MHz, CDCl_3_) *δ* 151.30, 150.15, 141.83, 141.11, 139.29, 136.76, 135.62, 133.60, 130.97, 130.75, 129.12, 128.64, 127.70, 127.24, 127.17, 125.86, 125.19, 112.46, 41.45, 40.52

; HR-ESI-MS: *m/z*: [M+H]^+^ calcd for C_25_H_27_N_4_, 383.2236; found, 383.2229.

**8-benzyl-6-(4-(dimethylamino)phenyl)-2-(4-hydroxybenzyl)imidazo[1,2-a]pyrazin-3(7H)-one (1a)**

The aminopyrazine derivative **7a** (30 mg, 0.1 mmol) and ketoacetal **9** (36 mg, 0.15 mmol) were dissolved in ethanol (1.0 mL), 12 M hydrochloric acid (100 μL) was added, and the mixture was stirred at 60 ° C. for 12 hours. After completion of the reaction, the reaction mixture was concentrated under reduced pressure. The obtained residue was separated by column chromatography (CHCl_3_ / MeOH = 10/1 to 5/1) to give CTZ analog **1a** (20 mg, 0.080 mmol, 80%) as a brown solid.

; ^1^H-NMR (500 MHz, CD_3_OD) *δ* 7.54 (s, 1 H), 7.44 (d, *J* = 6.9 Hz, 2 H), 7.38 (d, *J* = 7.4 Hz, 2 H), 7.28–7.31 (m, 2 H), 7.23 (t, *J* = 7.4 Hz, 1 H), 7.16 (d, *J* = 8.6 Hz, 2 H), 6.79 (d, *J* = 9.2 Hz, 2 H), 6.69 (dd, *J* = 8.9, 2.6 Hz, 2 H), 4.40 (s, 2H), 4.06 (s, 2 H), 2.97 (d, *J* = 12.0 Hz, 6 H)

; ^13^C-NMR (125 MHz, CD_3_OD) *δ* 155.66, 151.51, 136.76, 129.47, 129.28, 128.45, 128.39, 127.26, 126.86, 114.85, 112.05, 105.63, 72.19, 60.92, 39.07

; HR-ESI-MS: *m/z*: [M+H]^+^ calcd for C_28_H_27_N_4_O_2_, 451.2134; found, 451.2130, [M+Na]^+^ calcd for C_28_H_26_N_4_O_2_Na, 473.1947; found, 473.1953.

CTZ analogues **1b-d** were prepared with a similar procedure to the preparation of **1a**.

**(*E*)-8-benzyl-6-(4-(dimethylamino)styryl)-2-(4-hydroxybenzyl)imidazo[1,2-a]pyrazin-3(7H)-one (1b)**

(yield 78%), brown crystals:

; ^1^H-NMR (500 MHz, CD_3_OD) *δ* 7.44 (s, 1H), 7.36 (dd, *J* = 11.5, 8.0 Hz, 2H), 7.30 (dd, *J* = 11.5, 8.0 Hz, 2H), 7.27 (d, *J* = 6.3 Hz, 2H), 7.21 (t, *J* = 7.2 Hz, 1H), 7.15 (d, *J* = 8.6 Hz, 2H), 7.08 (d, *J* = 16.0 Hz, 1H), 6.69 (d, *J* = 8.6 Hz, 2H), 6.66 (d, *J* = 8.6 Hz, 2H), 6.60 (d, *J* = 16.6 Hz, 1H), 4.35 (s, 2H), 4.03 (s, 2H), 2.92 (s, 6H); HR-ESI-MS: *m/z*: [M+H]^+^ calcd for C_30_H_29_N_4_O_2_, 477.2291; found, 477.2287, [M+Na]^+^ calcd for C_30_H_28_N_4_O_2_Na, 499.2104; found, 499.2094.

**8-benzyl-6-((1*E*,3*E*)-4-(4-(dimethylamino)phenyl)buta-1,3-dien-1-yl)-2-(4-hydroxybenzyl)imidazo[1,2-a]pyrazin-3(7H)-one (1c)**

(yield 40%), brown crystals:

; ^1^H-NMR (500 MHz, CD_3_OD) *δ* 7.47 (s, 1 H), 7.36 (d, *J* = 8.0 Hz, 2 H), 7.27–7.33 (m, 4 H), 7.23 (t, *J* = 7.4 Hz, 1 H), 7.14 (d, *J* = 8.6 Hz, 2 H), 7.02–7.09 (m, 1 H), 6.77 (dd, *J* = 15.2, 10.6 Hz, 1 H), 6.68–6.71 (m, 4 H), 6.64 (d, *J* = 15.0 Hz, 1 H), 6.33 (d, *J* = 15.5 Hz, 1 H), 4.38 (s, 2 H), 4.03 (s, 2 H), 2.95 (s, 6 H)

; HR-ESI-MS: *m/z*: [M+H]^+^ calcd for C_32_H_31_N_4_O_2_, 503.2447; found, 503.2463, [M+Na]^+^ calcd for C_32_H_30_N_4_O_2_Na, 525.2266; found, 525.2263.

**8-benzyl-6-((1*E*,3*E*,5*E*)-6-(4-(dimethylamino)phenyl)hexa-1,3,5-trien-1-yl)-2-(4-hydroxybenzyl)imidazo[1,2-a]pyrazin-3(7H)-one (1d)**

(yield 28%), brown crystals:

; ^1^H-NMR (500 MHz, CD_3_OD) *δ* 7.44 (s, 1H), 7.33 (d, *J* = 7.4 Hz, 2H), 7.29-7.26 (m, 4H), 7.21 (t, *J* = 7.4 Hz, 1H), 7.12 (d, *J* = 8.6 Hz, 2H), 6.70-6.65 (m, 6H), 6.56-6.51 (m, 2H), 6.38 (dd, *J* = 14.6, 10.6 Hz, 1H), 6.28 (d, *J* = 15.5 Hz, 1H), 4.35 (s, 2H), 4.01 (s, 2H), 2.92 (s, 6H)

; HR-ESI-MS: *m/z*: [M+H]^+^ calcd for C_34_H_33_N_4_O_2_, 529.2604; found, 529.2637, [M+Na]^+^ calcd for C_34_H_32_N_4_O_2_Na, 551.2423; found, 551.2409, [M+K]^+^ calcd for C_34_H_32_N_4_O_2_K, 567.2162; found, 567.2144.

**3-phenyl-5-(4-(dimethylamino)phenyl)pyrazin-2-amine** (**8a**)

2-amino-3-phenyl-5-bromopyrazine **4b** (80.1 mg, 0.303 mmol), 4- (dimethylamino) phenylboronic acid (51.5 mg, 0.312 mmol), tetrakis (triphenylphosphine) palladium (0) (19.3 mg, 0.017 mmol) in 1,4 -Dissolved in dioxane (1 mL), added 1M aqueous sodium carbonate solution (1 mL) under an argon atmosphere, and stirred at 90 ° C for 3.5 hours. After completion of the reaction, the reaction mixture was extracted with ethyl acetate (2 × 50 mL), and washed with saturated saline. The organic layers were combined, dried over sodium sulfate, and then concentrated under reduced pressure. The obtained residue was separated by flash chromatography (hexane / ethyl acetate = 2 / 1) to give 2-amino-3-phenyl-5-dimethylaminophenylpyrazine **8a** (64.2 mg, 0.22 mmol, 74%) as yellow crystals.

; ^1^H-NMR (500 MHz, CDCl_3_) δ 7.97 (s, 1H), 7.82-7.87 (m, 4H), 7.42-7.52 (m, 3H), 6.79 (d, J = 9.2 Hz, 2H), 4.66 (s, 2H) , 2.98 (s, 6H)

; MS (ESI) : m/z 291.13 ([M+H]+)

Aminopyrazine derivative **8b-d** were synthesized from the pinacol borane ester **6a-c** in the similar procedure to the preparation of **8a**.

**(E)-3-phenyl-5-(4-(dimethylamino)styryl)pyrazin-2-amine (8b)**

(yield 30%), yellow crystals:

; 1H-NMR (500 MHz, CDCl_3_) δ 8.04 (s, 1H), 7.78 (d, J = 6.9 Hz, 2H), 7.42-7.53 (m, 7H), 4.83 (s, 1H), 4.11 (d, J = 6.9 Hz, 1H), 4.12 (s, 2H), 2.99 (s, 6H)

; MS (ESI) : m/z 317.15 ([M+H]+)

**3-phenyl-5-((1E,3E)-4-(4-(dimethylamino)phenyl)buta-1,3-dien-1-yl)pyrazin-2-amine (8c)**

(yield 80%), yellow crystals:

; 1H-NMR (500 MHz, CDCl_3_) δ 7.97 (s, 1H), 7.77 (d, J = 6.9 Hz, 2H), 7.45-7.53 (m, 3H), 7.29-7.35 (m, 3H), 7.04 (s, 1H), 6.79 (d, J = 10.9 Hz, 1H), 6.62 (dd, J = 35.2, 15.2 Hz, 4H), 4.74 (s, 1H), 2.98 (s, 6H)

; MS (ESI) : m/z 343.17 ([M+H]+)

**3-phenyl-5-((1E,3E,5E)-6-(4-(dimethylamino)phenyl)hexa-1,3,5-trien-1-yl)pyrazin-2-amine (8d)** (yield 37%), yellow crystals:

; 1H-NMR (500 MHz, CDCl_3_) δ 7.94 (s, 1H), 7.64-7.86 (m, 3H), 7.39-7.56 (m, 4H), 7.27-7.35 (m, 1H), 6.60-6.74 (m, 3H), 6.47-6.60 (m, 2H), 6.34-6.47 (m, 2H), 4.67-4.85 (m, 2H), 2.78-3.10 (m, 6H)

; MS (ESI) : m/z 369.19 ([M+H]+)

**8-phenyl-6-(4-(dimethylamino)phenyl)-2-(4-benzyl)imidazo[1,2-a]pyrazin-3(7H)-one (2a)**

The aminopyrazine derivative **8a** (15 mg, 0.05 mmol) and ketoacetal **10** (22 mg, 0.15 mmol) were dissolved in ethanol (1.0 mL), 12 M hydrochloric acid (100 μL) was added, and the mixture was stirred at 60 ° C. for 12 hours. After completion of the reaction, the reaction mixture was concentrated under reduced pressure. The obtained residue was separated by column chromatography (CHCl_3_ / MeOH = 15/1 to 10/1 to 5/1) to give CTZ analog **2a** (2.0 mg, 0.005 mmol, 10%) as a brown solid.

; 1H-NMR (500 MHz, CDCl_3_) *δ* 8.01 (s, 1H), 7.59-7.55 (m, 4H), 7.47 (t, *J* = 4.0 Hz, 2H), 7.42-7.40 (m, 2H), 7.36-7.35 (m, 1H), 7.29 (d, *J* = 8.0 Hz, 2H), 7.18 (t, *J* = 7.4 Hz, 1H), 6.77 (d, *J* = 9.2 Hz, 2H), 4.23 (s, 2H), 3.02 (s, 6H)

; MS (ESI) : *m/z* 421.20 ([M+H]^+^).

CTZ analogues **2b-d** were prepared with a similar procedure to the preparation of **2a**.

**(*E*)-8-phenyl-6-(4-(dimethylamino)styryl)-2-(4-benzyl)imidazo[1,2-a]pyrazin-3(7H)-one (2b)**

(yield 13%), brown crystals:

; ^1^H-NMR (500 MHz, CD_3_OD) *δ* 7.98 (s, 1H), 7.88 (d, *J* = 6.9 Hz, 2H), 7.58-7.52 (m, 4H), 7.38 (s, 2H), 7.29 (d, *J* = 7.4 Hz, 2H), 7.19 (t, *J* = 7.4 Hz, 2H), 7.11 (t, *J* = 7.2 Hz, 1H), 6.97 (d, *J* = 6.9 Hz, 2H), 6.82 (d, *J* = 14.9 Hz, 1H), 4.13 (s, 2H), 3.34 (s, 6H)

; MS (ESI) : *m/z* 447.22 ([M+H]^+^)

**8-phenyl-6-((1*E*,3*E*)-4-(4-(dimethylamino)phenyl)buta-1,3-dien-1-yl)-2-(4-benzyl)imidazo[1,2-a]pyrazin-3(7H)-one (2c)**

(yield 20%), brown crystals:

; ^1^H-NMR (500 MHz, CD_3_OD) *δ* 8.07 (s, 1H), 7.90-7.90 (m, 3H), 7.72 (d, *J* = 7.4 Hz, 1H), 7.57-7.52 (m, 4H), 7.36-7.31 (m, 3H), 7.23 (t, *J* = 7.7 Hz, 1H), 7.18-7.15 (m, 2H), 6.84 (dd, *J* = 15.5, 10.3 Hz, 1H), 6.74-6.69 (m, 3H), 2.96 (s, 6H), 2.85 (s, 2H)

; MS (ESI) : *m/z* 473.23 ([M+H]^+^)

**8-phenyl-6-((1*E*,3*E*,5*E*)-6-(4-(dimethylamino)phenyl)hexa-1,3,5-trien-1-yl)-2-(4-benzyl)imidazo[1,2-a]pyrazin-3(7H)-one (2d)**

(yield 25%), brown crystals:

; ^1^H-NMR (500 MHz, CD_3_OD) *δ* 8.00 (d, *J* = 5.7 Hz, 2H), 7.57-7.55 (m, 4H), 7.30 (d, *J* = 8.6 Hz, 4H), 7.22 (t, *J* = 7.7 Hz, 2H), 7.12 (t, *J* = 7.4 Hz, 1H), 6.77-6.69 (m, 4H), 6.62-6.54 (m, 2H), 6.46 (dd, *J* = 15.2, 10.6 Hz, 2H), 4.10 (s, 2H), 2.94 (s, 6H)

; MS (ESI) : *m/z* 499.29 ([M+H]^+^)

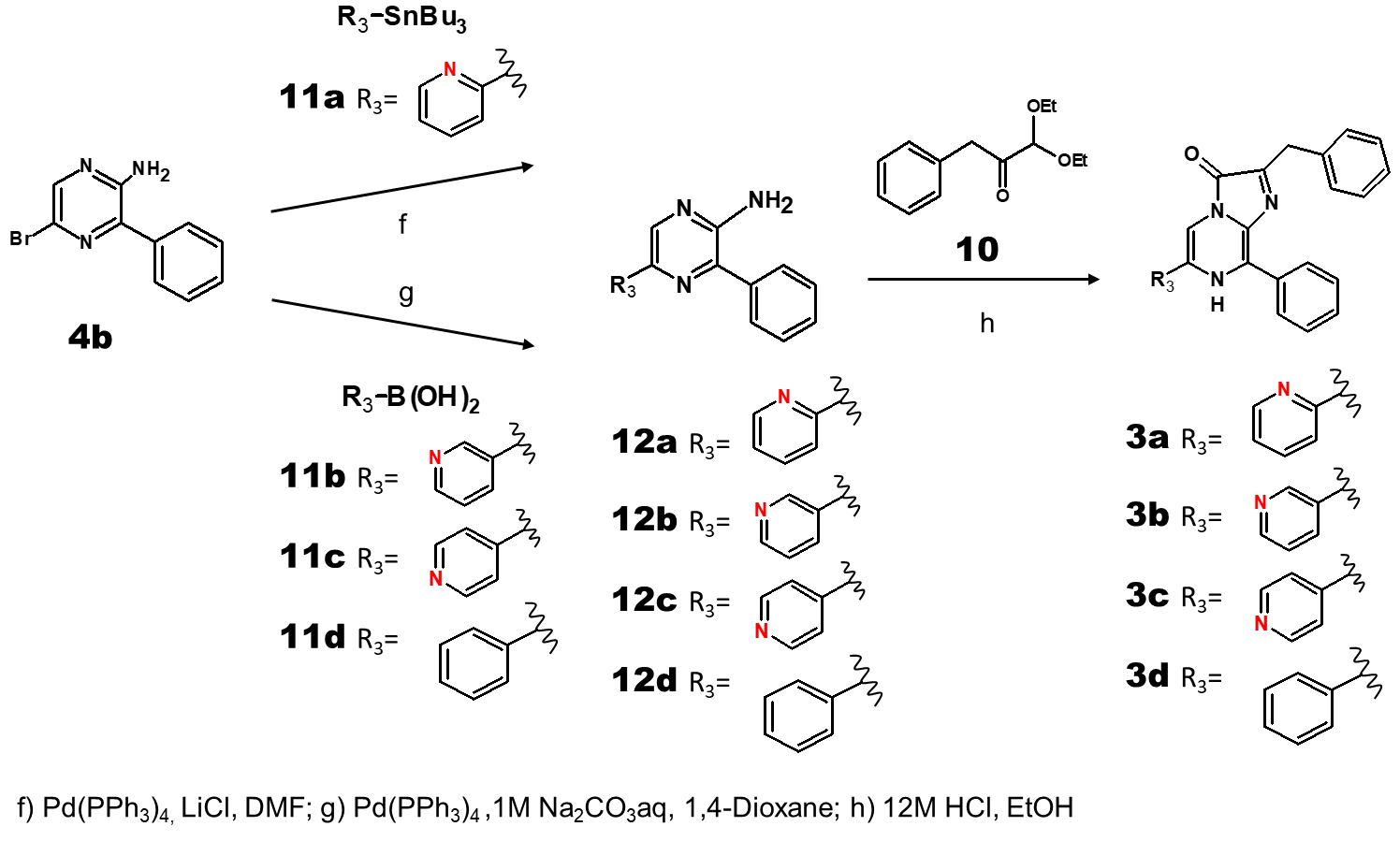


**Figure S9.** Synthetic scheme for **3a-d**

(compound **3d, 12d** are known procedures)

**3-phenyl-5-(pyridin-2-yl)pyrazin-2-amine (11a)**

　2-amino-3-phenyl-5-bromopyrazine (**4b**) (501.9 mg, 2.00 mmol), 2- (tributyltin) pyridine (865.0 mg, 2.35 mmol), tetrakistriphenylphosphine palladium (127.0 mg, 0.11 mol) Then, lithium chloride (135.8 mg, 3.20 mmol) was dissolved in 30 mL of DMF, and the mixture was heated under reflux at 90 ° C. for 40 minutes under an argon atmosphere. After completion of the reaction, the reaction mixture was concentrated under reduced pressure using an evaporator equipped with a vacuum trap. The obtained yellow solid substance was roughly purified by silica gel column chromatography containing 10% potassium carbonate (hexane / ethyl acetate = 1/1). The crude product was separated by silica gel column chromatography (hexane / ethyl acetate = 1 / 1 → ethyl acetate) to obtain an aminopyrazine derivative **11a** (333.6 mg, 1.34 mmol, 67.2%) as yellow crystals.

; ^1^H-NMR (500 MHz, CDCl_3_) *δ* 9.06 (s, 1H), 8.62-8.64 (m, 1H), 8.27 (d, *J* = 8.0 Hz, 1H), 7.81-7.84 (m, 2H), 7.74 (qd, *J* = 7.3, 1.7 Hz, 1H), 7.53 (td, *J* = 6.6, 1.7 Hz, 3H), 7.44-7.50 (m, 2H), 4.90 (d, *J* = 40.7 Hz, 2H)

; MS (ESI) : *m/z* 249.10 ([M+H]^+^)

**3-phenyl-5-(pyridin-3-yl)pyrazin-2-amine (11b)**

2-amino-3-phenyl-5-bromopyrazine (**4b**) (126.1 mg, 0.50 mmol), 3-pyridineboronic acid (69.0 mg, 0.56 mmol), tetrakistriphenylphosphine palladium (45.9 mg, 0.04 mol), The mixture was dissolved in 24 mL of 1,4-dioxane, 15 mL of a 1 M aqueous solution of sodium carbonate was added under an argon atmosphere, and the mixture was heated under reflux at 100 ° C. for 4 hours. After the reaction was completed, 50 mL of water was added, and the product was extracted with ethyl acetate (2 × 50 mL). The organic phase was washed with saturated saline, dried over anhydrous sodium sulfate, and then concentrated under reduced pressure. The obtained yellow solid substance was separated by column chromatography (hexane / ethyl acetate = 1 / 1 → ethyl acetate) to obtain an aminopyrazine derivative **11b** (130.3 mg, 0.52 mmol, 101.9%) as a yellow crystals.

; 1H-NMR (500 MHz, CDCl_3_) *δ* 9.18 (d, *J* = 2.3 Hz, 1H), 8.59 (dd, *J* = 4.9, 1.4 Hz, 1H), 8.27 (d, *J* = 8.0 Hz, 1H), 7.80-7.82 (m, 2H), 7.54 (t, *J* = 7.4 Hz, 2H), 7.44-7.49 (m, 2H), 7.36 (dd, *J* = 7.7, 4.9 Hz, 1H), 4.92 (s, 2H)

; MS (ESI) : *m/z* 249.10 ([M+H]^+^)

Aminopyrazine derivative **11c** were synthesized in the similar procedure to the preparation of **11b**.

**3-phenyl-5-(pyridin-4-yl)pyrazin-2-amine (11c)**

; ^1^H-NMR (500 MHz, CDCl_3_) *δ* 8.54 (s, 1H), 7.90 (d, *J* = 5.7 Hz, 2H), 7.79-7.81 (m, 2H), 7.51-7.56 (m, 2H), 7.44-7.51 (m, 3H), 5.02 (s, 2H)

; MS (ESI) : *m/z* 249.11 ([M+H]^+^)

**2-benzyl-8-phenyl-6-(pyridin-2-yl)imidazo[1,2-a]pyrazin-3(7H)-one (3a)**

The aminopyrazine derivative **8a** (94 mg, 0.4 mmol) and ketoacetal **10** (101 mg, 0.4 mmol) were dissolved in ethanol (11 mL), 12 M hydrochloric acid (100 μL) was added, and the mixture was stirred at 60 ° C. for 12 hours. After completion of the reaction, the reaction mixture was concentrated under reduced pressure. The obtained residue was separated by column chromatography (CHCl_3_ / MeOH = 20/1 to 15/1 to 10/1) to give CTZ analog **2a** (63 mg, 0.16 mmol, 42%) as a red crystals.

; ^1^H-NMR (500 MHz, CD_3_OD) *δ* 8.65 (d, *J* = 4.6 Hz, 1H), 8.23-8.18 (m, 3H), 7.93 (td, *J* = 7.7, 1.7 Hz, 1H), 7.62-7.59 (m, 3H), 7.41 (dd, *J* = 7.2, 5.4 Hz, 1H), 7.34 (d, *J* = 7.4 Hz, 2H), 7.26 (t, *J* = 7.7 Hz, 2H), 7.16 (t, *J* = 7.4 Hz, 1H), 4.18 (s, 2H)

; MS (ESI) : *m/z* 379.15 ([M+H]^+^)

CTZ analogues **3b-c** were prepared with a similar procedure to the preparation of **3a**.

**2-benzyl-8-phenyl-6-(pyridin-3-yl)imidazo[1,2-a]pyrazin-3(7H)-one (3b)**

(yield 41%), red crystals:

; ^1^H-NMR (500 MHz, CD_3_OD) *δ* 9.11 (d, *J* = 1.7 Hz, 1H), 8.54 (dd, *J* = 4.9, 1.4 Hz, 1H), 8.35 (d, *J* = 8.0 Hz, 1H), 8.24 (s, 1H), 8.16-8.14 (m, 2H), 7.55-7.52 (m, 3H), 7.32 (d, *J* = 7.4 Hz, 2H), 7.24 (t, *J* = 7.7 Hz, 2H), 7.15 (t, *J* = 7.2 Hz, 1H), 4.15 (s, 2H)

; MS (ESI) : *m/z* 379.11 ([M+H]^+^)

**2-benzyl-8-phenyl-6-(pyridin-4-yl)imidazo[1,2-a]pyrazin-3(7H)-one (3c)**

(yield 18%), red crystals:

; ^1^H-NMR (500 MHz, CD_3_OD) *δ* 8.61 (dd, *J* = 4.6, 1.7 Hz, 2H), 8.50 (s, 1H), 8.24-8.22 (m, 2H), 8.06 (d, *J* = 6.3 Hz, 2H), 7.55-7.53 (m, 3H), 7.31 (d, *J* = 7.4 Hz, 2H), 7.24 (t, *J* = 7.7 Hz, 2H), 7.14 (t, *J* = 7.4 Hz, 1H), 4.16 (s, 2H)

; MS (ESI) : *m/z* 379.13 ([M+H]^+^)

1. **Computational methods**
   1. **DFT and TD DFT calculations of oxy-1a’-d’, oxy-2a’-d’, oxy-1a’-d’(an), and oxy-2a’-d’(an).**

Density functional theory (DFT) calculations were performed with the Gaussian 09 program (Rev. D.01).^5^ DFT includes the B3LYP function with the 6-31+G(d) basis set^6-8^ and IEF-PCM approximation in DMSO.^9^ The molecular structures of oxy-**1a**’-**d**’, oxy-**2a**’-**d**’, oxy-**1a**’-**d**’(an), and oxy-**2a**’-**d**’(an) were optimized by DFT calculations as shown in Figure S10. Based on their optimized structures, time-dependent (TD) DFT calculations were carried out to give the properties of the electronic transitions including the S_0_→S_1_ transitions. Molecular graphics were made with GaussView, Version 5.^10^

- 1. **Heats of formation.**

**Table S1.** Heats of formation of oxy-**1a**’-**d**’, oxy-**2a**’-**d**’, oxy-**1a**’-**d**’(an), and oxy-**2a**’-**d**’(an) with DFT using B3LYP/6-31+G(d) (IEF-PCM, DMSO).

| Compound | Heat of formation/hartree | Compound | Heat of formation/hartree |
| --- | --- | --- | --- |
| oxy-**1a**’ | −1107.76888898 | oxy-**1a**’(an) | −1107.28767139 |
| oxy-**1b**’ | −1185.17727352 | oxy-**1b**’(an) | −1184.69681071 |
| oxy-**1c**’ | −1262.58690091 | oxy-**1c**’(an) | −1262.10691394 |
| oxy-**1d**’ | −1339.99680515 | oxy-**1d**’(an) | −1339.51742138 |
| oxy-**2a**’ | −1068.45599861 | oxy-**2a**’(an) | −1067.97435822 |
| oxy-**2b**’ | −1145.86436264 | oxy-**2b**’(an) | −1145.38320218 |
| oxy-**2c**’ | −1223.27400210 | oxy-**2c**’(an) | −1222.79335953 |
| oxy-**2d**’ | −1300.68391854 | oxy-**2d**’(an) | −1300.20356437 |

- 1. **Optimized structures.**

**Figure S10.** Optimized structures of oxy-**1a**’-**d**’, oxy-**2a**’-**d**’, oxy-**1a**’-**d**’(an), and oxy-**2a**’-**d**’(an).

- 1. **Geometries of the compounds optimized with DFT using B3LYP/6-31+G(d) (IEF-PCM, DMSO).**

**Table S2**. Cartesian Coordinates (in Å) of oxy-**1a**’.

|  |  | *x* | *y* | *z* |
| --- | --- | --- | --- | --- |
| 1 | H | 2.886414 | -3.005617 | -6.726107 |
| 2 | H | 1.366736 | -3.115371 | -7.647239 |
| 3 | H | 2.789217 | -2.319569 | -8.352465 |
| 4 | H | 0.944635 | 0.360429 | -8.072228 |
| 5 | H | 2.446476 | 0.822669 | -7.235454 |
| 6 | H | 2.511925 | -0.253828 | -8.636699 |
| 7 | H | 1.248586 | -3.231306 | -5.239895 |
| 8 | H | 0.098429 | -3.087614 | -3.129476 |
| 9 | H | -0.364414 | 1.162001 | -3.629347 |
| 10 | H | 0.794629 | 1.046075 | -5.764916 |
| 11 | H | -0.304231 | -2.756491 | -1.053757 |
| 12 | H | -3.91459 | -0.239786 | 1.623442 |
| 13 | N | 1.833746 | -1.176268 | -6.892687 |
| 14 | C | 2.232544 | -2.472762 | -7.427168 |
| 15 | C | 1.932529 | 0.000314 | -7.74835 |
| 16 | C | 1.155498 | -1.102448 | -5.697405 |
| 17 | C | 0.92068 | -2.255631 | -4.901644 |
| 18 | C | 0.259931 | -2.166566 | -3.681888 |
| 19 | C | -0.216399 | -0.939288 | -3.179392 |
| 20 | C | -0.00351 | 0.200579 | -3.979724 |
| 21 | C | 0.658654 | 0.132284 | -5.198455 |
| 22 | C | -0.91286 | -0.829693 | -1.883646 |
| 23 | C | -0.876203 | -1.845528 | -0.906288 |
| 24 | N | -1.536561 | -1.749196 | 0.247024 |
| 25 | C | -2.214027 | -0.623717 | 0.479925 |
| 26 | C | -2.232684 | 0.434211 | -0.455528 |
| 27 | N | -1.599265 | 0.302545 | -1.621243 |
| 28 | N | -2.946256 | -0.528896 | 1.691009 |
| 29 | C | -2.434022 | -0.725875 | 2.952289 |
| 30 | O | -1.242028 | -0.955495 | 3.161762 |
| 31 | C | -3.445965 | -0.650159 | 4.079233 |
| 32 | H | -4.401521 | -0.210021 | 3.780666 |
| 33 | H | -3.633529 | -1.664766 | 4.449909 |
| 34 | H | -3.020025 | -0.067802 | 4.900848 |
| 35 | C | -2.994916 | 1.720436 | -0.166617 |
| 36 | H | -2.861856 | 1.963859 | 0.894539 |
| 37 | H | -4.06898 | 1.522397 | -0.295441 |
| 38 | C | -2.601789 | 2.910587 | -1.01704 |
| 39 | C | -3.383624 | 3.305798 | -2.111382 |
| 40 | C | -1.451857 | 3.654974 | -0.710589 |
| 41 | C | -3.023416 | 4.412482 | -2.888731 |
| 42 | H | -4.282606 | 2.744856 | -2.358412 |
| 43 | C | -1.08641 | 4.760438 | -1.484348 |
| 44 | H | -0.838038 | 3.368068 | 0.140971 |
| 45 | C | -1.871523 | 5.142596 | -2.578682 |
| 46 | H | -3.643773 | 4.704049 | -3.732665 |
| 47 | H | -0.192899 | 5.325495 | -1.230388 |
| 48 | H | -1.59077 | 6.004123 | -3.179183 |

**Table S3**. Cartesian Coordinates (in Å) of oxy-**1b**’.

|  |  | *x* | *y* | *z* |
| --- | --- | --- | --- | --- |
| 1 | H | 4.304133 | -0.414681 | -8.96646 |
| 2 | H | 2.965468 | -1.362721 | -9.657576 |
| 3 | H | 3.780822 | -0.13655 | -10.638587 |
| 4 | H | 1.212902 | 1.964358 | -10.296776 |
| 5 | H | 2.636358 | 2.7455 | -9.567222 |
| 6 | H | 2.82088 | 1.697282 | -10.986483 |
| 7 | H | 2.780806 | -1.325017 | -7.405967 |
| 8 | H | 1.70161 | -1.47818 | -5.250646 |
| 9 | H | -0.297021 | 2.27266 | -5.958609 |
| 10 | H | 0.778413 | 2.45086 | -8.131117 |
| 11 | H | 0.095277 | -2.463123 | -1.171369 |
| 12 | H | -3.741988 | -0.420252 | 1.626152 |
| 13 | N | 2.478737 | 0.653161 | -9.212603 |
| 14 | C | 3.430378 | -0.368692 | -9.632407 |
| 15 | C | 2.274243 | 1.827318 | -10.051968 |
| 16 | C | 1.874983 | 0.573367 | -7.980315 |
| 17 | C | 2.107741 | -0.529289 | -7.109365 |
| 18 | C | 1.486746 | -0.613016 | -5.872298 |
| 19 | C | 0.598327 | 0.384762 | -5.410641 |
| 20 | C | 0.376924 | 1.478815 | -6.274877 |
| 21 | C | 0.989293 | 1.582946 | -7.517629 |
| 22 | C | -0.5645 | -1.622974 | -0.964731 |
| 23 | N | -1.222901 | -1.645202 | 0.193094 |
| 24 | C | -2.025514 | -0.6137 | 0.461948 |
| 25 | C | -2.183387 | 0.461922 | -0.44639 |
| 26 | N | -1.540491 | 0.452094 | -1.61204 |
| 27 | N | -2.754449 | -0.638984 | 1.676819 |
| 28 | C | -2.226022 | -0.858003 | 2.928921 |
| 29 | O | -1.019489 | -0.996708 | 3.132352 |
| 30 | C | -3.241829 | -0.928153 | 4.05285 |
| 31 | H | -4.231669 | -0.55669 | 3.772985 |
| 32 | H | -3.341798 | -1.973351 | 4.368541 |
| 33 | H | -2.867955 | -0.355854 | 4.906265 |
| 34 | C | -3.107964 | 1.62675 | -0.119038 |
| 35 | H | -3.017489 | 1.843011 | 0.952348 |
| 36 | H | -4.145914 | 1.296815 | -0.272617 |
| 37 | C | -2.862203 | 2.891713 | -0.914889 |
| 38 | C | -3.679694 | 3.233509 | -2.001092 |
| 39 | C | -1.815917 | 3.759015 | -0.563458 |
| 40 | C | -3.45462 | 4.409107 | -2.726614 |
| 41 | H | -4.499838 | 2.576183 | -2.282469 |
| 42 | C | -1.585825 | 4.933806 | -1.285159 |
| 43 | H | -1.177152 | 3.513727 | 0.282751 |
| 44 | C | -2.405023 | 5.262594 | -2.371854 |
| 45 | H | -4.100462 | 4.657489 | -3.565251 |
| 46 | H | -0.771772 | 5.594144 | -0.996463 |
| 47 | H | -2.230167 | 6.177642 | -2.931983 |
| 48 | C | -0.716738 | -0.580226 | -1.901234 |
| 49 | C | 0.005486 | -0.615345 | -3.167323 |
| 50 | C | -0.081283 | 0.342866 | -4.124549 |
| 51 | H | 0.643185 | -1.485315 | -3.310306 |
| 52 | H | -0.737118 | 1.187959 | -3.91867 |

**Table S4**. Cartesian Coordinates (in Å) of oxy-**1c**’.

|  |  | *x* | *y* | *z* |
| --- | --- | --- | --- | --- |
| 1 | H | 4.202618 | -0.255283 | -9.547782 |
| 2 | H | 2.855519 | -1.134707 | -10.310368 |
| 3 | H | 3.791507 | 0.051805 | -11.240267 |
| 4 | H | 1.059149 | 1.850776 | -11.17513 |
| 5 | H | 2.286012 | 2.938945 | -10.482598 |
| 6 | H | 2.736004 | 1.792709 | -11.753136 |
| 7 | H | 2.787026 | -0.930729 | -7.938963 |
| 8 | H | 1.672143 | -0.970617 | -5.797339 |
| 9 | H | -0.484868 | 2.615105 | -6.827042 |
| 10 | H | 0.624654 | 2.676758 | -8.98822 |
| 11 | H | -0.599624 | -2.365464 | 0.660094 |
| 12 | H | -4.622135 | -0.237228 | 3.143863 |
| 13 | N | 2.466003 | 0.91467 | -9.879831 |
| 14 | C | 3.370777 | -0.16446 | -10.257735 |
| 15 | C | 2.11269 | 1.926197 | -10.868389 |
| 16 | C | 1.820879 | 0.883912 | -8.665682 |
| 17 | C | 2.078422 | -0.142627 | -7.712953 |
| 18 | C | 1.435613 | -0.162449 | -6.484276 |
| 19 | C | 0.493206 | 0.824934 | -6.114548 |
| 20 | C | 0.236701 | 1.836241 | -7.066345 |
| 21 | C | 0.869763 | 1.874566 | -8.302285 |
| 22 | C | -1.319992 | -1.555948 | 0.757388 |
| 23 | N | -2.004047 | -1.49287 | 1.899329 |
| 24 | C | -2.883428 | -0.499015 | 2.032592 |
| 25 | C | -3.100873 | 0.446619 | 0.998101 |
| 26 | N | -2.425475 | 0.353811 | -0.143476 |
| 27 | N | -3.631406 | -0.427863 | 3.231703 |
| 28 | C | -3.124641 | -0.510252 | 4.510416 |
| 29 | O | -1.920808 | -0.598817 | 4.750872 |
| 30 | C | -4.163818 | -0.49414 | 5.614954 |
| 31 | H | -5.152132 | -0.16495 | 5.282143 |
| 32 | H | -4.256778 | -1.507799 | 6.022418 |
| 33 | H | -3.816303 | 0.15896 | 6.42003 |
| 34 | C | -4.12133 | 1.563724 | 1.174256 |
| 35 | H | -3.980116 | 1.997966 | 2.172311 |
| 36 | H | -5.12634 | 1.117032 | 1.182301 |
| 37 | C | -4.074336 | 2.659108 | 0.129776 |
| 38 | C | -4.955078 | 2.653429 | -0.961179 |
| 39 | C | -3.156462 | 3.714193 | 0.245794 |
| 40 | C | -4.915234 | 3.671476 | -1.920471 |
| 41 | H | -5.678965 | 1.847276 | -1.061684 |
| 42 | C | -3.111401 | 4.733329 | -0.710172 |
| 43 | H | -2.472077 | 3.738528 | 1.091434 |
| 44 | C | -3.991167 | 4.714479 | -1.798873 |
| 45 | H | -5.607225 | 3.650314 | -2.758783 |
| 46 | H | -2.394499 | 5.543553 | -0.602345 |
| 47 | H | -3.960595 | 5.508132 | -2.54105 |
| 48 | C | -1.517512 | -0.638715 | -0.293645 |
| 49 | C | -0.7556 | -0.7544 | -1.528451 |
| 50 | H | -0.061746 | -1.592781 | -1.579467 |
| 51 | C | -0.850646 | 0.095196 | -2.587298 |
| 52 | H | -1.545583 | 0.931578 | -2.518777 |
| 53 | C | -0.091766 | -0.03144 | -3.802932 |
| 54 | H | 0.597008 | -0.873363 | -3.868289 |
| 55 | C | -0.208042 | 0.844639 | -4.841162 |
| 56 | H | -0.911228 | 1.670103 | -4.717515 |

**Table S5**. Cartesian Coordinates (in Å) of oxy-**1d**’.

|  |  | *x* | *y* | *z* |
| --- | --- | --- | --- | --- |
| 1 | H | 5.11794 | 0.106035 | -11.702931 |
| 2 | H | 3.851175 | -1.011542 | -12.265038 |
| 3 | H | 4.610964 | 0.105559 | -13.404769 |
| 4 | H | 2.018075 | 2.234061 | -13.354304 |
| 5 | H | 3.441229 | 3.058507 | -12.672622 |
| 6 | H | 3.631664 | 1.874035 | -13.981845 |
| 7 | H | 3.535935 | -0.732964 | -10.087739 |
| 8 | H | 2.402447 | -0.664652 | -7.959318 |
| 9 | H | 0.342786 | 2.930479 | -9.144294 |
| 10 | H | 1.472429 | 2.883882 | -11.293729 |
| 11 | H | -0.643009 | -2.422911 | 0.801444 |
| 12 | H | -4.626312 | -0.089198 | 3.175804 |
| 13 | N | 3.233642 | 1.009782 | -12.125303 |
| 14 | C | 4.256015 | 0.001483 | -12.37885 |
| 15 | C | 3.070957 | 2.103742 | -13.075118 |
| 16 | C | 2.602498 | 1.06117 | -10.903369 |
| 17 | C | 2.836295 | 0.074075 | -9.904383 |
| 18 | C | 2.183423 | 0.117162 | -8.681625 |
| 19 | C | 1.260135 | 1.138656 | -8.358846 |
| 20 | C | 1.040138 | 2.120564 | -9.34991 |
| 21 | C | 1.684672 | 2.096076 | -10.580514 |
| 22 | C | -1.3447 | -1.594084 | 0.867608 |
| 23 | N | -2.035957 | -1.479291 | 2.001254 |
| 24 | C | -2.891763 | -0.461069 | 2.095663 |
| 25 | C | -3.078235 | 0.457437 | 1.02977 |
| 26 | N | -2.397231 | 0.312123 | -0.102505 |
| 27 | N | -3.649434 | -0.33326 | 3.282421 |
| 28 | C | -3.172259 | -0.424854 | 4.572846 |
| 29 | O | -1.980983 | -0.577203 | 4.840301 |
| 30 | C | -4.232745 | -0.33364 | 5.653463 |
| 31 | H | -5.183825 | 0.074278 | 5.299441 |
| 32 | H | -4.415661 | -1.33917 | 6.050769 |
| 33 | H | -3.854339 | 0.285713 | 6.470966 |
| 34 | C | -4.070212 | 1.605781 | 1.164284 |
| 35 | H | -3.917091 | 2.073098 | 2.145593 |
| 36 | H | -5.086489 | 1.185809 | 1.18825 |
| 37 | C | -3.995784 | 2.659756 | 0.079803 |
| 38 | C | -4.863953 | 2.623568 | -1.020621 |
| 39 | C | -3.064042 | 3.705479 | 0.165669 |
| 40 | C | -4.797943 | 3.602021 | -2.018734 |
| 41 | H | -5.598073 | 1.824187 | -1.098097 |
| 42 | C | -2.993199 | 4.685466 | -0.829058 |
| 43 | H | -2.389077 | 3.753353 | 1.017833 |
| 44 | C | -3.860295 | 4.63599 | -1.926824 |
| 45 | H | -5.480245 | 3.557466 | -2.86404 |
| 46 | H | -2.266021 | 5.48925 | -0.74434 |
| 47 | H | -3.809473 | 5.399141 | -2.699258 |
| 48 | C | -1.512571 | -0.706668 | -0.213201 |
| 49 | C | -0.744785 | -0.882003 | -1.436444 |
| 50 | H | -0.070414 | -1.737473 | -1.45172 |
| 51 | C | -0.810634 | -0.069917 | -2.527302 |
| 52 | H | -1.485304 | 0.785054 | -2.497518 |
| 53 | C | -0.044425 | -0.265076 | -3.726493 |
| 54 | H | 0.626574 | -1.124948 | -3.760187 |
| 55 | C | -0.113936 | 0.560888 | -4.813727 |
| 56 | H | -0.786095 | 1.420111 | -4.771546 |
| 57 | C | 0.63947 | 0.381216 | -6.023598 |
| 58 | C | 0.552044 | 1.224884 | -7.092264 |
| 59 | H | 1.305246 | -0.480802 | -6.060229 |
| 60 | H | -0.128462 | 2.073526 | -7.002346 |

**Table S6**. Cartesian Coordinates (in Å) of oxy-**1a**’(an).

|  |  | *x* | *y* | *z* |
| --- | --- | --- | --- | --- |
| 1 | H | 3.490494 | -2.500957 | -6.416018 |
| 2 | H | 2.083985 | -3.225908 | -7.232639 |
| 3 | H | 3.210595 | -2.179101 | -8.127893 |
| 4 | H | 0.471541 | -0.559647 | -8.293893 |
| 5 | H | 1.517147 | 0.757444 | -7.709105 |
| 6 | H | 2.171061 | -0.455784 | -8.810515 |
| 7 | H | 2.193497 | -2.811282 | -4.742653 |
| 8 | H | 1.047077 | -2.653984 | -2.627739 |
| 9 | H | -1.101241 | 0.803797 | -4.007901 |
| 10 | H | 0.047951 | 0.677676 | -6.146613 |
| 11 | H | -0.639307 | -2.882885 | -1.227669 |
| 12 | N | 1.943907 | -1.126032 | -6.848527 |
| 13 | C | 2.713277 | -2.323064 | -7.167063 |
| 14 | C | 1.493794 | -0.306699 | -7.967663 |
| 15 | C | 1.247871 | -1.070375 | -5.647767 |
| 16 | C | 1.476949 | -2.008988 | -4.610375 |
| 17 | C | 0.806722 | -1.91984 | -3.39219 |
| 18 | C | -0.139062 | -0.91064 | -3.130908 |
| 19 | C | -0.373263 | 0.013858 | -4.165564 |
| 20 | C | 0.287376 | -0.059567 | -5.388967 |
| 21 | C | -0.862925 | -0.81415 | -1.84286 |
| 22 | C | -1.021545 | -1.897015 | -0.970621 |
| 23 | N | -1.664771 | -1.807597 | 0.200361 |
| 24 | C | -2.194118 | -0.614734 | 0.550729 |
| 25 | C | -2.065648 | 0.492067 | -0.356943 |
| 26 | N | -1.415364 | 0.378489 | -1.506464 |
| 27 | N | -2.951769 | -0.472547 | 1.685907 |
| 28 | C | -2.505694 | -0.838288 | 2.891062 |
| 29 | O | -1.319156 | -1.14636 | 3.211105 |
| 30 | C | -3.551091 | -0.814956 | 4.004833 |
| 31 | H | -4.537717 | -0.513626 | 3.64155 |
| 32 | H | -3.626319 | -1.812726 | 4.455728 |
| 33 | H | -3.234972 | -0.127157 | 4.799374 |
| 34 | C | -2.687909 | 1.827266 | 0.026379 |
| 35 | H | -2.246019 | 2.145677 | 0.980141 |
| 36 | H | -3.747298 | 1.648441 | 0.252588 |
| 37 | C | -2.555082 | 2.934837 | -0.994967 |
| 38 | C | -3.532717 | 3.12174 | -1.9845 |
| 39 | C | -1.460862 | 3.813107 | -0.968244 |
| 40 | C | -3.417473 | 4.148867 | -2.927353 |
| 41 | H | -4.394213 | 2.457324 | -2.016319 |
| 42 | C | -1.339122 | 4.842867 | -1.907308 |
| 43 | H | -0.696082 | 3.690118 | -0.203717 |
| 44 | C | -2.317885 | 5.013884 | -2.892885 |
| 45 | H | -4.18813 | 4.276525 | -3.683948 |
| 46 | H | -0.483246 | 5.512489 | -1.86675 |
| 47 | H | -2.22798 | 5.815657 | -3.621578 |

**Table S7**. Cartesian Coordinates (in Å) of oxy-**1b**’(an).

|  |  | *x* | *y* | *z* |
| --- | --- | --- | --- | --- |
| 1 | H | 4.299898 | -0.421413 | -8.980201 |
| 2 | H | 2.964459 | -1.420331 | -9.604075 |
| 3 | H | 3.738407 | -0.234532 | -10.65726 |
| 4 | H | 1.438512 | 2.223549 | -10.16033 |
| 5 | H | 3.019344 | 2.646419 | -9.458991 |
| 6 | H | 2.921769 | 1.696925 | -10.959939 |
| 7 | H | 2.598552 | -1.42991 | -7.471016 |
| 8 | H | 1.535169 | -1.5363 | -5.308855 |
| 9 | H | -0.098941 | 2.403674 | -5.89145 |
| 10 | H | 0.963469 | 2.535723 | -8.070716 |
| 11 | H | -0.232589 | -2.580552 | -1.356068 |
| 12 | N | 2.44132 | 0.598859 | -9.252576 |
| 13 | C | 3.412101 | -0.420601 | -9.632781 |
| 14 | C | 2.457 | 1.858732 | -9.986001 |
| 15 | C | 1.862777 | 0.55351 | -7.994715 |
| 16 | C | 2.003096 | -0.582381 | -7.151983 |
| 17 | C | 1.389032 | -0.640362 | -5.906753 |
| 18 | C | 0.604261 | 0.421116 | -5.40594 |
| 19 | C | 0.485823 | 1.553408 | -6.237917 |
| 20 | C | 1.092733 | 1.630888 | -7.488464 |
| 21 | C | -0.751442 | -1.670037 | -1.055078 |
| 22 | N | -1.349479 | -1.69142 | 0.141367 |
| 23 | C | -2.007471 | -0.580114 | 0.541588 |
| 24 | C | -2.040509 | 0.564715 | -0.337271 |
| 25 | N | -1.440152 | 0.558539 | -1.515508 |
| 26 | N | -2.735809 | -0.550842 | 1.698351 |
| 27 | C | -2.249714 | -0.968606 | 2.873744 |
| 28 | O | -1.040657 | -1.199307 | 3.16504 |
| 29 | C | -3.283393 | -1.108247 | 3.989926 |
| 30 | H | -4.29426 | -0.860104 | 3.654134 |
| 31 | H | -3.275372 | -2.139242 | 4.365979 |
| 32 | H | -3.014186 | -0.457792 | 4.831703 |
| 33 | C | -2.791284 | 1.807324 | 0.11906 |
| 34 | H | -2.393767 | 2.099765 | 1.099583 |
| 35 | H | -3.83256 | 1.516792 | 0.314005 |
| 36 | C | -2.750255 | 2.987327 | -0.825873 |
| 37 | C | -3.73383 | 3.155721 | -1.812624 |
| 38 | C | -1.736572 | 3.95305 | -0.726811 |
| 39 | C | -3.702429 | 4.250513 | -2.683119 |
| 40 | H | -4.534139 | 2.423254 | -1.89932 |
| 41 | C | -1.698374 | 5.050137 | -1.593945 |
| 42 | H | -0.96947 | 3.84551 | 0.037733 |
| 43 | C | -2.681916 | 5.202329 | -2.577904 |
| 44 | H | -4.476409 | 4.362043 | -3.438843 |
| 45 | H | -0.904616 | 5.787244 | -1.49797 |
| 46 | H | -2.65709 | 6.056224 | -3.250349 |
| 47 | C | -0.774343 | -0.560439 | -1.908725 |
| 48 | C | -0.09571 | -0.599412 | -3.201588 |
| 49 | C | -0.063714 | 0.41004 | -4.105399 |
| 50 | H | 0.414374 | -1.537949 | -3.416049 |
| 51 | H | -0.59514 | 1.326168 | -3.849991 |

**Table S8**. Cartesian Coordinates (in Å) of oxy-**1c**’(an).

|  |  | *x* | *y* | *z* |
| --- | --- | --- | --- | --- |
| 1 | H | 4.512149 | 0.23245 | -9.469334 |
| 2 | H | 3.253285 | -0.88336 | -10.053066 |
| 3 | H | 3.983647 | 0.275428 | -11.166338 |
| 4 | H | 1.506515 | 2.584549 | -10.905865 |
| 5 | H | 3.02694 | 3.184869 | -10.200364 |
| 6 | H | 3.05107 | 2.119337 | -11.62399 |
| 7 | H | 2.847936 | -0.765587 | -7.926773 |
| 8 | H | 1.725722 | -0.80026 | -5.791613 |
| 9 | H | -0.162403 | 2.963075 | -6.696687 |
| 10 | H | 0.957338 | 3.020982 | -8.850008 |
| 11 | H | -0.800076 | -2.45698 | 0.453252 |
| 12 | N | 2.605697 | 1.12017 | -9.847909 |
| 13 | C | 3.64278 | 0.137362 | -10.139142 |
| 14 | C | 2.544641 | 2.31711 | -10.678019 |
| 15 | C | 1.992408 | 1.119596 | -8.607999 |
| 16 | C | 2.185626 | 0.056944 | -7.683321 |
| 17 | C | 1.538484 | 0.040485 | -6.454467 |
| 18 | C | 0.664946 | 1.075112 | -6.051592 |
| 19 | C | 0.492248 | 2.136003 | -6.965652 |
| 20 | C | 1.13162 | 2.170921 | -8.200886 |
| 21 | C | -1.420644 | -1.580658 | 0.642649 |
| 22 | N | -2.095406 | -1.565055 | 1.796723 |
| 23 | C | -2.882316 | -0.495083 | 2.057974 |
| 24 | C | -2.936547 | 0.581499 | 1.095689 |
| 25 | N | -2.26336 | 0.53625 | -0.040707 |
| 26 | N | -3.710061 | -0.455373 | 3.143111 |
| 27 | C | -3.314607 | -0.788522 | 4.379546 |
| 28 | O | -2.128192 | -0.915738 | 4.795046 |
| 29 | C | -4.443027 | -0.961099 | 5.394393 |
| 30 | H | -5.426807 | -0.766958 | 4.958063 |
| 31 | H | -4.42411 | -1.986037 | 5.786809 |
| 32 | H | -4.286199 | -0.288295 | 6.246679 |
| 33 | C | -3.809958 | 1.790997 | 1.396329 |
| 34 | H | -3.692436 | 2.031957 | 2.459025 |
| 35 | H | -4.861494 | 1.488127 | 1.2874 |
| 36 | C | -3.539165 | 3.018364 | 0.553865 |
| 37 | C | -4.335219 | 3.329579 | -0.558159 |
| 38 | C | -2.486926 | 3.888491 | 0.88267 |
| 39 | C | -4.084652 | 4.471862 | -1.32758 |
| 40 | H | -5.160432 | 2.67212 | -0.824911 |
| 41 | C | -2.230337 | 5.030688 | 0.118448 |
| 42 | H | -1.863145 | 3.66866 | 1.747079 |
| 43 | C | -3.029166 | 5.326555 | -0.992732 |
| 44 | H | -4.715459 | 4.694237 | -2.185037 |
| 45 | H | -1.411507 | 5.691643 | 0.392718 |
| 46 | H | -2.834472 | 6.216186 | -1.586499 |
| 47 | C | -1.487577 | -0.550341 | -0.304395 |
| 48 | C | -0.743678 | -0.631184 | -1.554295 |
| 49 | H | -0.135077 | -1.527809 | -1.678475 |
| 50 | C | -0.754 | 0.293768 | -2.551492 |
| 51 | H | -1.366125 | 1.186616 | -2.426421 |
| 52 | C | -0.007897 | 0.172038 | -3.78021 |
| 53 | H | 0.602376 | -0.724887 | -3.89217 |
| 54 | C | -0.039609 | 1.101554 | -4.774476 |
| 55 | H | -0.667541 | 1.980347 | -4.616169 |

**Table S9**. Cartesian Coordinates (in Å) of oxy-**1d**’(an).

|  |  | *x* | *y* | *z* |
| --- | --- | --- | --- | --- |
| 1 | H | 5.36927 | 0.256226 | -11.568483 |
| 2 | H | 3.955441 | -0.602145 | -12.228022 |
| 3 | H | 4.89379 | 0.472301 | -13.267818 |
| 4 | H | 2.830075 | 3.143851 | -12.926401 |
| 5 | H | 4.407704 | 3.450877 | -12.160255 |
| 6 | H | 4.297959 | 2.467209 | -13.637313 |
| 7 | H | 3.53144 | -0.52206 | -10.101195 |
| 8 | H | 2.378243 | -0.471012 | -7.983791 |
| 9 | H | 1.152953 | 3.591862 | -8.722434 |
| 10 | H | 2.307171 | 3.564662 | -10.857365 |
| 11 | H | -1.110992 | -2.660247 | 0.461972 |
| 12 | N | 3.64676 | 1.469033 | -11.92522 |
| 13 | C | 4.511558 | 0.341765 | -12.254304 |
| 14 | C | 3.803968 | 2.698833 | -12.692586 |
| 15 | C | 3.014039 | 1.509892 | -10.696558 |
| 16 | C | 3.010354 | 0.386833 | -9.824213 |
| 17 | C | 2.346315 | 0.420117 | -8.605177 |
| 18 | C | 1.648943 | 1.565939 | -8.160423 |
| 19 | C | 1.668109 | 2.681791 | -9.024419 |
| 20 | C | 2.328193 | 2.668328 | -10.248693 |
| 21 | C | -1.610971 | -1.715339 | 0.676574 |
| 22 | N | -2.246022 | -1.628674 | 1.849602 |
| 23 | C | -2.884678 | -0.471215 | 2.141011 |
| 24 | C | -2.85936 | 0.603219 | 1.173206 |
| 25 | N | -2.219269 | 0.491422 | 0.023443 |
| 26 | N | -3.643272 | -0.328729 | 3.265158 |
| 27 | C | -3.228931 | -0.710039 | 4.481941 |
| 28 | O | -2.046637 | -0.977975 | 4.837347 |
| 29 | C | -4.316295 | -0.744758 | 5.553619 |
| 30 | H | -5.295636 | -0.458552 | 5.160274 |
| 31 | H | -4.381867 | -1.757527 | 5.971292 |
| 32 | H | -4.049265 | -0.073649 | 6.379597 |
| 33 | C | -3.599576 | 1.891307 | 1.502044 |
| 34 | H | -3.236415 | 2.25127 | 2.473392 |
| 35 | H | -4.65347 | 1.634041 | 1.673848 |
| 36 | C | -3.496705 | 2.994262 | 0.472391 |
| 37 | C | -4.435289 | 3.10261 | -0.565373 |
| 38 | C | -2.469733 | 3.948351 | 0.54218 |
| 39 | C | -4.346329 | 4.127491 | -1.513525 |
| 40 | H | -5.244591 | 2.377924 | -0.631054 |
| 41 | C | -2.374489 | 4.975797 | -0.402342 |
| 42 | H | -1.73775 | 3.887275 | 1.345304 |
| 43 | C | -3.313136 | 5.06831 | -1.436218 |
| 44 | H | -5.085494 | 4.193568 | -2.308408 |
| 45 | H | -1.571582 | 5.705423 | -0.327937 |
| 46 | H | -3.244168 | 5.868166 | -2.169295 |
| 47 | C | -1.572041 | -0.673203 | -0.259003 |
| 48 | C | -0.856248 | -0.823678 | -1.516798 |
| 49 | H | -0.374691 | -1.790727 | -1.667309 |
| 50 | C | -0.74183 | 0.12061 | -2.490899 |
| 51 | H | -1.222032 | 1.086544 | -2.337248 |
| 52 | C | -0.020113 | -0.071881 | -3.721154 |
| 53 | H | 0.456734 | -1.042715 | -3.871858 |
| 54 | C | 0.102917 | 0.875327 | -4.697683 |
| 55 | H | -0.373552 | 1.845933 | -4.544821 |
| 56 | C | 0.825303 | 0.690553 | -5.929263 |
| 57 | C | 0.941725 | 1.651014 | -6.888891 |
| 58 | H | 1.296183 | -0.282373 | -6.073196 |
| 59 | H | 0.453866 | 2.608878 | -6.698643 |

**Table S10**. Cartesian Coordinates (in Å) of oxy-**2a**’.

|  |  | *x* | *y* | *z* |
| --- | --- | --- | --- | --- |
| 1 | H | 3.422126 | -2.343704 | -6.500438 |
| 2 | H | 2.012779 | -3.237457 | -7.120535 |
| 3 | H | 2.950259 | -2.178339 | -8.195276 |
| 4 | H | 0.210346 | -0.453269 | -8.211008 |
| 5 | H | 1.397271 | 0.775963 | -7.711244 |
| 6 | H | 1.868575 | -0.49344 | -8.846665 |
| 7 | H | 2.156281 | -2.743968 | -4.738929 |
| 8 | H | 1.157724 | -2.532225 | -2.558844 |
| 9 | H | -1.098287 | 0.886387 | -3.883527 |
| 10 | H | -0.103851 | 0.696475 | -6.09414 |
| 11 | H | -0.272591 | -2.680682 | -0.964005 |
| 12 | N | 1.719365 | -1.140004 | -6.869022 |
| 13 | C | 2.564105 | -2.286846 | -7.181052 |
| 14 | C | 1.268586 | -0.284176 | -7.960209 |
| 15 | C | 1.131853 | -1.037382 | -5.627555 |
| 16 | C | 1.44517 | -1.942282 | -4.578564 |
| 17 | C | 0.860036 | -1.820575 | -3.323454 |
| 18 | C | -0.070084 | -0.803944 | -3.029484 |
| 19 | C | -0.381261 | 0.09315 | -4.070047 |
| 20 | C | 0.189104 | -0.015752 | -5.331785 |
| 21 | C | -0.703604 | -0.6716 | -1.703857 |
| 22 | C | -0.710808 | -1.710489 | -0.75036 |
| 23 | N | -1.239127 | -1.563612 | 0.46283 |
| 24 | C | -1.837636 | -0.405307 | 0.748132 |
| 25 | C | -1.912779 | 0.635673 | -0.210161 |
| 26 | N | -1.31758 | 0.488884 | -1.400136 |
| 27 | C | -2.995019 | -1.131725 | 2.841921 |
| 28 | O | -3.303511 | -2.258071 | 2.454237 |
| 29 | C | -3.319 | -0.650144 | 4.243606 |
| 30 | H | -3.223644 | 0.432167 | 4.368667 |
| 31 | H | -4.337096 | -0.956576 | 4.49862 |
| 32 | H | -2.635302 | -1.139141 | 4.947726 |
| 33 | C | -2.598444 | 1.935352 | 0.04085 |
| 34 | C | -1.951499 | 3.136314 | -0.299839 |
| 35 | C | -3.899737 | 1.991815 | 0.5707 |
| 36 | C | -2.585104 | 4.365221 | -0.103024 |
| 37 | H | -0.948505 | 3.099508 | -0.714797 |
| 38 | C | -4.536582 | 3.221986 | 0.756903 |
| 39 | H | -4.425576 | 1.073757 | 0.817139 |
| 40 | C | -3.880082 | 4.411999 | 0.425788 |
| 41 | H | -2.068263 | 5.285318 | -0.362902 |
| 42 | H | -5.547269 | 3.248541 | 1.15539 |
| 43 | H | -4.374437 | 5.368089 | 0.575932 |
| 44 | N | -2.325166 | -0.21548 | 2.059205 |
| 45 | H | -2.269819 | 0.730628 | 2.416581 |

**Table S11**. Cartesian Coordinates (in Å) of oxy-**2b**’.

|  |  | *x* | *y* | *z* |
| --- | --- | --- | --- | --- |
| 1 | H | 3.042889 | -1.919206 | -6.655874 |
| 2 | H | 1.537434 | -2.476735 | -7.425712 |
| 3 | H | 2.708732 | -1.506608 | -8.341717 |
| 4 | H | 0.354505 | 0.729987 | -8.27507 |
| 5 | H | 1.733094 | 1.623031 | -7.58821 |
| 6 | H | 2.005631 | 0.410949 | -8.846057 |
| 7 | H | 1.597344 | -2.261181 | -5.015486 |
| 8 | H | 0.536932 | -2.07814 | -2.855638 |
| 9 | H | -0.934946 | 1.838769 | -3.882753 |
| 10 | H | 0.121487 | 1.678268 | -6.063948 |
| 11 | H | -1.133124 | -2.501917 | 1.276984 |
| 12 | N | 1.602907 | -0.396014 | -6.964626 |
| 13 | C | 2.250384 | -1.641178 | -7.361293 |
| 14 | C | 1.407042 | 0.646023 | -7.965317 |
| 15 | C | 0.977041 | -0.301121 | -5.742789 |
| 16 | C | 1.053733 | -1.352502 | -4.785485 |
| 17 | C | 0.442125 | -1.243838 | -3.545581 |
| 18 | C | -0.29069 | -0.095463 | -3.169113 |
| 19 | C | -0.374181 | 0.938451 | -4.126282 |
| 20 | C | 0.230606 | 0.849668 | -5.374275 |
| 21 | C | -1.647837 | -1.55564 | 1.430725 |
| 22 | N | -2.222336 | -1.37098 | 2.616979 |
| 23 | C | -2.87571 | -0.224155 | 2.816996 |
| 24 | C | -2.946755 | 0.770329 | 1.805019 |
| 25 | N | -2.319349 | 0.584752 | 0.63937 |
| 26 | C | -4.076415 | -0.890814 | 4.906887 |
| 27 | O | -4.314786 | -2.051493 | 4.57559 |
| 28 | C | -4.474068 | -0.347393 | 6.266429 |
| 29 | H | -4.455331 | 0.744603 | 6.32469 |
| 30 | H | -5.476375 | -0.706977 | 6.513883 |
| 31 | H | -3.780273 | -0.743215 | 7.01779 |
| 32 | C | -3.667926 | 2.063977 | 1.979204 |
| 33 | C | -3.030932 | 3.263436 | 1.615478 |
| 34 | C | -4.989545 | 2.114601 | 2.456556 |
| 35 | C | -3.694724 | 4.485934 | 1.738405 |
| 36 | H | -2.01208 | 3.23086 | 1.240723 |
| 37 | C | -5.656281 | 3.337993 | 2.56856 |
| 38 | H | -5.507369 | 1.196473 | 2.719231 |
| 39 | C | -5.010019 | 4.52708 | 2.214997 |
| 40 | H | -3.185617 | 5.405422 | 1.461678 |
| 41 | H | -6.681866 | 3.359568 | 2.927276 |
| 42 | H | -5.527834 | 5.478042 | 2.307513 |
| 43 | N | -3.423715 | 0.013124 | 4.09502 |
| 44 | H | -3.42711 | 0.978987 | 4.399977 |
| 45 | C | -1.663512 | -0.57434 | 0.418497 |
| 46 | C | -0.980565 | -0.811435 | -0.847775 |
| 47 | C | -0.948667 | 0.067706 | -1.880808 |
| 48 | H | -0.477428 | -1.773199 | -0.924027 |
| 49 | H | -1.473501 | 1.012576 | -1.743304 |

**Table S12**. Cartesian Coordinates (in Å) of oxy-**2c**’.

|  |  | *x* | *y* | *z* |
| --- | --- | --- | --- | --- |
| 1 | H | 3.455666 | -1.780736 | -7.700819 |
| 2 | H | 1.956296 | -2.35398 | -8.47111 |
| 3 | H | 3.137987 | -1.407443 | -9.39886 |
| 4 | H | 0.774958 | 0.819602 | -9.406713 |
| 5 | H | 2.144904 | 1.738616 | -8.737041 |
| 6 | H | 2.431158 | 0.493754 | -9.959135 |
| 7 | H | 2.004772 | -2.078014 | -6.061722 |
| 8 | H | 0.929003 | -1.841813 | -3.91493 |
| 9 | H | -0.541398 | 2.045642 | -5.053095 |
| 10 | H | 0.530409 | 1.830607 | -7.221039 |
| 11 | H | -1.579509 | -2.657009 | 2.540773 |
| 12 | N | 2.02181 | -0.263004 | -8.058354 |
| 13 | C | 2.670264 | -1.51828 | -8.419885 |
| 14 | C | 1.826125 | 0.749833 | -9.088678 |
| 15 | C | 1.387147 | -0.138286 | -6.843428 |
| 16 | C | 1.45852 | -1.164426 | -5.85893 |
| 17 | C | 0.838195 | -1.025337 | -4.626346 |
| 18 | C | 0.100923 | 0.131518 | -4.283689 |
| 19 | C | 0.022659 | 1.140242 | -5.268788 |
| 20 | C | 0.636274 | 1.020287 | -6.509472 |
| 21 | C | -2.086264 | -1.699906 | 2.646486 |
| 22 | N | -2.687594 | -1.464074 | 3.809965 |
| 23 | C | -3.331654 | -0.302856 | 3.950157 |
| 24 | C | -3.36314 | 0.655344 | 2.901479 |
| 25 | N | -2.710635 | 0.418737 | 1.759401 |
| 26 | C | -4.609296 | -0.881176 | 6.018754 |
| 27 | O | -4.865176 | -2.044285 | 5.710218 |
| 28 | C | -5.032993 | -0.292044 | 7.350887 |
| 29 | H | -4.996818 | 0.800736 | 7.377182 |
| 30 | H | -6.047266 | -0.627822 | 7.58229 |
| 31 | H | -4.365358 | -0.676258 | 8.131455 |
| 32 | C | -4.069362 | 1.964281 | 3.010975 |
| 33 | C | -3.406203 | 3.140359 | 2.618824 |
| 34 | C | -5.40107 | 2.0507 | 3.453947 |
| 35 | C | -4.054489 | 4.375656 | 2.680813 |
| 36 | H | -2.37962 | 3.079485 | 2.269631 |
| 37 | C | -6.052156 | 3.286511 | 3.504398 |
| 38 | H | -5.93827 | 1.150153 | 3.737603 |
| 39 | C | -5.380087 | 4.452582 | 3.123353 |
| 40 | H | -3.525458 | 5.277147 | 2.383061 |
| 41 | H | -7.085504 | 3.335655 | 3.837165 |
| 42 | H | -5.885855 | 5.41341 | 3.168398 |
| 43 | N | -3.911605 | -0.014683 | 5.203011 |
| 44 | H | -3.906083 | 0.960124 | 5.478136 |
| 45 | C | -2.066297 | -0.757299 | 1.597873 |
| 46 | C | -1.363266 | -1.048947 | 0.357204 |
| 47 | H | -0.861339 | -2.014929 | 0.316451 |
| 48 | C | -1.300219 | -0.22077 | -0.72058 |
| 49 | H | -1.810429 | 0.740606 | -0.666024 |
| 50 | C | -0.602893 | -0.526834 | -1.941218 |
| 51 | H | -0.092036 | -1.488341 | -1.98787 |
| 52 | C | -0.565144 | 0.324121 | -3.005665 |
| 53 | H | -1.094944 | 1.272862 | -2.902676 |

**Table S13**. Cartesian Coordinates (in Å) of oxy-**2d**’.

|  |  | *x* | *y* | *z* |
| --- | --- | --- | --- | --- |
| 1 | H | 2.985547 | 0.707243 | -9.52635 |
| 2 | H | 1.461674 | 0.13208 | -10.245381 |
| 3 | H | 2.508944 | 1.25297 | -11.138967 |
| 4 | H | 0.070363 | 3.35986 | -10.692904 |
| 5 | H | 1.460731 | 4.236484 | -10.00881 |
| 6 | H | 1.687741 | 3.169717 | -11.40066 |
| 7 | H | 1.652383 | 0.112078 | -7.847218 |
| 8 | H | 0.716881 | 0.017628 | -5.624284 |
| 9 | H | -1.002395 | 3.933416 | -6.157045 |
| 10 | H | -0.070422 | 4.051908 | -8.396807 |
| 11 | H | -2.006232 | -2.507944 | 3.112711 |
| 12 | N | 1.444447 | 2.160116 | -9.59117 |
| 13 | C | 2.130449 | 1.000157 | -10.147958 |
| 14 | C | 1.144003 | 3.289396 | -10.462941 |
| 15 | C | 0.893535 | 2.098066 | -8.331781 |
| 16 | C | 1.079693 | 0.961685 | -7.494181 |
| 17 | C | 0.539779 | 0.910717 | -6.217721 |
| 18 | C | -0.225268 | 1.973531 | -5.683597 |
| 19 | C | -0.414772 | 3.09359 | -6.523076 |
| 20 | C | 0.117548 | 3.164435 | -7.804159 |
| 21 | C | -2.528215 | -1.583342 | 3.350507 |
| 22 | N | -3.065328 | -1.49289 | 4.564808 |
| 23 | C | -3.726777 | -0.373391 | 4.86779 |
| 24 | C | -3.848288 | 0.689199 | 3.931556 |
| 25 | N | -3.258406 | 0.59742 | 2.736317 |
| 26 | C | -4.845261 | -1.199516 | 6.946802 |
| 27 | O | -5.087049 | -2.336025 | 6.542772 |
| 28 | C | -5.198652 | -0.75665 | 8.354005 |
| 29 | H | -5.161001 | 0.327208 | 8.495462 |
| 30 | H | -6.199879 | -1.120288 | 8.600509 |
| 31 | H | -4.492296 | -1.219531 | 9.053379 |
| 32 | C | -4.583452 | 1.953902 | 4.221055 |
| 33 | C | -3.980332 | 3.18703 | 3.916645 |
| 34 | C | -5.88656 | 1.946991 | 4.749279 |
| 35 | C | -4.658477 | 4.385717 | 4.147859 |
| 36 | H | -2.97637 | 3.198771 | 3.502509 |
| 37 | C | -6.567974 | 3.147301 | 4.969742 |
| 38 | H | -6.379266 | 1.003729 | 4.9672 |
| 39 | C | -5.954827 | 4.369545 | 4.675081 |
| 40 | H | -4.17542 | 5.331287 | 3.916057 |
| 41 | H | -7.579458 | 3.125396 | 5.366444 |
| 42 | H | -6.484017 | 5.302068 | 4.852197 |
| 43 | N | -4.230226 | -0.2336 | 6.177466 |
| 44 | H | -4.231919 | 0.708619 | 6.549298 |
| 45 | C | -2.59174 | -0.532078 | 2.412825 |
| 46 | C | -1.949445 | -0.661079 | 1.113488 |
| 47 | H | -1.419495 | -1.596713 | 0.939089 |
| 48 | C | -1.971852 | 0.283045 | 0.132664 |
| 49 | H | -2.510222 | 1.211679 | 0.320185 |
| 50 | C | -1.331648 | 0.14134 | -1.145271 |
| 51 | H | -0.786771 | -0.78444 | -1.336636 |
| 52 | C | -1.373362 | 1.099313 | -2.119936 |
| 53 | H | -1.922908 | 2.020687 | -1.917061 |
| 54 | C | -0.744573 | 0.989176 | -3.406774 |
| 55 | H | -0.194853 | 0.069684 | -3.606841 |
| 56 | C | -0.813256 | 1.968229 | -4.354702 |
| 57 | H | -1.376936 | 2.867467 | -4.099904 |

**Table S14**. Cartesian Coordinates (in Å) of oxy-**2a**’(an).

|  |  | *x* | *y* | *z* |
| --- | --- | --- | --- | --- |
| 1 | H | 4.044342 | -0.809046 | -6.032612 |
| 2 | H | 3.758719 | -2.562206 | -6.155036 |
| 3 | H | 3.930828 | -1.569315 | -7.620925 |
| 4 | H | 0.945782 | -2.61847 | -7.924984 |
| 5 | H | 0.623426 | -0.878085 | -8.119192 |
| 6 | H | 2.126576 | -1.564786 | -8.737619 |
| 7 | H | 3.080515 | -1.217695 | -4.148902 |
| 8 | H | 1.805105 | -1.071859 | -2.10569 |
| 9 | H | -1.863039 | -1.225644 | -4.342948 |
| 10 | H | -0.604329 | -1.36434 | -6.418246 |
| 11 | H | 0.55838 | -1.902429 | -0.455559 |
| 12 | N | 2.092617 | -1.323639 | -6.666154 |
| 13 | C | 3.526157 | -1.584558 | -6.607576 |
| 14 | C | 1.40675 | -1.617221 | -7.918938 |
| 15 | C | 1.363147 | -1.286884 | -5.486557 |
| 16 | C | 1.999244 | -1.223028 | -4.220973 |
| 17 | C | 1.259834 | -1.146904 | -3.042882 |
| 18 | C | -0.147478 | -1.143062 | -3.043284 |
| 19 | C | -0.778073 | -1.216112 | -4.29919 |
| 20 | C | -0.055539 | -1.293425 | -5.486297 |
| 21 | C | -0.937772 | -1.068408 | -1.793844 |
| 22 | C | -0.433355 | -1.463377 | -0.544686 |
| 23 | N | -1.122493 | -1.335777 | 0.59083 |
| 24 | C | -2.37867 | -0.831203 | 0.53924 |
| 25 | C | -2.940955 | -0.532263 | -0.748543 |
| 26 | N | -2.210435 | -0.62657 | -1.863352 |
| 27 | N | -3.023621 | -0.48172 | 1.69988 |
| 28 | C | -3.318593 | -1.368264 | 2.648948 |
| 29 | O | -3.253321 | -2.632655 | 2.562589 |
| 30 | C | -3.849233 | -0.776388 | 3.952754 |
| 31 | H | -3.891704 | 0.31618 | 3.925161 |
| 32 | H | -4.852913 | -1.169763 | 4.158515 |
| 33 | H | -3.205772 | -1.087657 | 4.785497 |
| 34 | C | -4.349037 | -0.078351 | -0.920981 |
| 35 | C | -4.647925 | 0.948633 | -1.834377 |
| 36 | C | -5.410268 | -0.68505 | -0.225157 |
| 37 | C | -5.965998 | 1.366184 | -2.039117 |
| 38 | H | -3.836529 | 1.42155 | -2.380139 |
| 39 | C | -6.729614 | -0.277076 | -0.438551 |
| 40 | H | -5.201713 | -1.488562 | 0.473745 |
| 41 | C | -7.013611 | 0.753764 | -1.342208 |
| 42 | H | -6.174174 | 2.169225 | -2.742053 |
| 43 | H | -7.537243 | -0.7666 | 0.100351 |
| 44 | H | -8.039897 | 1.074737 | -1.502039 |

**Table S15**. Cartesian Coordinates (in Å) of oxy-**2b**’(an).

|  |  | *x* | *y* | *z* |
| --- | --- | --- | --- | --- |
| 1 | H | 2.968808 | -2.100696 | -6.623701 |
| 2 | H | 1.460461 | -2.313566 | -7.545792 |
| 3 | H | 2.853834 | -1.481694 | -8.27264 |
| 4 | H | 0.711809 | 0.896784 | -8.200133 |
| 5 | H | 2.072681 | 1.709218 | -7.388854 |
| 6 | H | 2.388731 | 0.544086 | -8.676763 |
| 7 | H | 1.494745 | -2.307261 | -5.055883 |
| 8 | H | 0.322109 | -2.153042 | -2.953333 |
| 9 | H | -0.669355 | 1.954293 | -3.7802 |
| 10 | H | 0.502871 | 1.823673 | -5.901911 |
| 11 | H | -1.495318 | -2.627505 | 1.098749 |
| 12 | N | 1.843572 | -0.325254 | -6.860977 |
| 13 | C | 2.296205 | -1.624125 | -7.345571 |
| 14 | C | 1.739765 | 0.761654 | -7.827007 |
| 15 | C | 1.136007 | -0.248373 | -5.673423 |
| 16 | C | 1.037625 | -1.360378 | -4.793306 |
| 17 | C | 0.36095 | -1.268082 | -3.583365 |
| 18 | C | -0.271435 | -0.076621 | -3.16576 |
| 19 | C | -0.187718 | 1.016793 | -4.052129 |
| 20 | C | 0.485073 | 0.944554 | -5.268558 |
| 21 | C | -1.98056 | -1.667362 | 1.276583 |
| 22 | N | -2.657842 | -1.542673 | 2.41777 |
| 23 | C | -3.277283 | -0.364549 | 2.678797 |
| 24 | C | -3.138852 | 0.710968 | 1.726587 |
| 25 | N | -2.475479 | 0.546068 | 0.582265 |
| 26 | C | -3.774495 | -0.581502 | 4.988368 |
| 27 | O | -2.601333 | -0.77984 | 5.422197 |
| 28 | C | -4.921833 | -0.648455 | 5.993794 |
| 29 | H | -5.890592 | -0.437905 | 5.532025 |
| 30 | H | -4.95196 | -1.647431 | 6.447146 |
| 31 | H | -4.748149 | 0.067712 | 6.807024 |
| 32 | C | -3.740901 | 2.056357 | 1.94675 |
| 33 | C | -4.336329 | 2.735993 | 0.868267 |
| 34 | C | -3.690719 | 2.70006 | 3.196401 |
| 35 | C | -4.875652 | 4.014558 | 1.033835 |
| 36 | H | -4.376353 | 2.250275 | -0.102409 |
| 37 | C | -4.21704 | 3.984421 | 3.35956 |
| 38 | H | -3.228251 | 2.197673 | 4.039156 |
| 39 | C | -4.816956 | 4.645713 | 2.281429 |
| 40 | H | -5.341008 | 4.516932 | 0.189191 |
| 41 | H | -4.157651 | 4.46971 | 4.330784 |
| 42 | H | -5.232719 | 5.641638 | 2.41231 |
| 43 | N | -4.12834 | -0.253546 | 3.74289 |
| 44 | C | -1.885881 | -0.6429 | 0.321147 |
| 45 | C | -1.15424 | -0.851117 | -0.926764 |
| 46 | C | -0.991831 | 0.074403 | -1.903101 |
| 47 | H | -0.719963 | -1.843883 | -1.037636 |
| 48 | H | -1.441707 | 1.05342 | -1.740403 |

**Table S16**. Cartesian Coordinates (in Å) of oxy-**2c**’(an).

|  |  | *x* | *y* | *z* |
| --- | --- | --- | --- | --- |
| 1 | H | 3.447437 | -1.871369 | -7.661784 |
| 2 | H | 1.944066 | -2.337732 | -8.493739 |
| 3 | H | 3.214733 | -1.444994 | -9.359089 |
| 4 | H | 0.857422 | 0.739111 | -9.428298 |
| 5 | H | 2.174758 | 1.727732 | -8.752071 |
| 6 | H | 2.539386 | 0.473175 | -9.939947 |
| 7 | H | 2.02783 | -2.076347 | -6.032495 |
| 8 | H | 0.908676 | -1.824537 | -3.910693 |
| 9 | H | -0.490217 | 2.075941 | -5.085172 |
| 10 | H | 0.624979 | 1.844525 | -7.228169 |
| 11 | H | -1.666653 | -2.666704 | 2.464672 |
| 12 | N | 2.127161 | -0.259856 | -8.030977 |
| 13 | C | 2.705032 | -1.546904 | -8.399822 |
| 14 | C | 1.904787 | 0.719807 | -9.087451 |
| 15 | C | 1.455371 | -0.130252 | -6.829448 |
| 16 | C | 1.48976 | -1.154763 | -5.843944 |
| 17 | C | 0.843576 | -1.006649 | -4.623564 |
| 18 | C | 0.113499 | 0.157982 | -4.296727 |
| 19 | C | 0.068908 | 1.163731 | -5.28531 |
| 20 | C | 0.708558 | 1.033902 | -6.513874 |
| 21 | C | -2.161495 | -1.704495 | 2.599742 |
| 22 | N | -2.753049 | -1.496533 | 3.775149 |
| 23 | C | -3.378504 | -0.310493 | 3.985101 |
| 24 | C | -3.415481 | 0.649344 | 2.905886 |
| 25 | N | -2.792057 | 0.421296 | 1.751055 |
| 26 | C | -4.619625 | -0.770849 | 5.958342 |
| 27 | O | -5.28407 | -1.768374 | 5.551619 |
| 28 | C | -4.778767 | -0.361013 | 7.420542 |
| 29 | H | -4.196365 | 0.531403 | 7.6663 |
| 30 | H | -5.836758 | -0.173164 | 7.643123 |
| 31 | H | -4.456371 | -1.186719 | 8.067604 |
| 32 | C | -4.127385 | 1.953412 | 3.022342 |
| 33 | C | -3.546216 | 3.114158 | 2.479777 |
| 34 | C | -5.396541 | 2.058235 | 3.619814 |
| 35 | C | -4.205626 | 4.344668 | 2.542557 |
| 36 | H | -2.568805 | 3.044339 | 2.011413 |
| 37 | C | -6.062878 | 3.285578 | 3.670671 |
| 38 | H | -5.866749 | 1.173671 | 4.035621 |
| 39 | C | -5.468849 | 4.435675 | 3.137816 |
| 40 | H | -3.732945 | 5.231374 | 2.127038 |
| 41 | H | -7.048324 | 3.343059 | 4.126569 |
| 42 | H | -5.985126 | 5.391202 | 3.185226 |
| 43 | N | -3.828032 | 0.022118 | 5.229982 |
| 44 | C | -2.141379 | -0.751817 | 1.567774 |
| 45 | C | -1.441875 | -1.02118 | 0.317835 |
| 46 | H | -0.944725 | -1.990278 | 0.262288 |
| 47 | C | -1.361129 | -0.183108 | -0.74989 |
| 48 | H | -1.860283 | 0.784009 | -0.691751 |
| 49 | C | -0.650316 | -0.487134 | -1.967704 |
| 50 | H | -0.151369 | -1.455913 | -2.010179 |
| 51 | C | -0.578884 | 0.362063 | -3.02933 |
| 52 | H | -1.095373 | 1.319563 | -2.938742 |

**Table S17**. Cartesian Coordinates (in Å) of oxy-**2d**’(an).

|  |  | *x* | *y* | *z* |
| --- | --- | --- | --- | --- |
| 1 | H | 2.955766 | 0.593404 | -9.524784 |
| 2 | H | 1.41193 | 0.099564 | -10.261813 |
| 3 | H | 2.52888 | 1.168045 | -11.138928 |
| 4 | H | 0.06867 | 3.207243 | -10.759237 |
| 5 | H | 1.397293 | 4.183325 | -10.086733 |
| 6 | H | 1.707338 | 3.089594 | -11.438378 |
| 7 | H | 1.689131 | 0.107536 | -7.814674 |
| 8 | H | 0.746372 | 0.054264 | -5.594677 |
| 9 | H | -0.979848 | 3.952835 | -6.209652 |
| 10 | H | -0.040892 | 4.029467 | -8.447065 |
| 11 | H | -1.966137 | -2.448017 | 3.089452 |
| 12 | N | 1.499802 | 2.12759 | -9.599151 |
| 13 | C | 2.124387 | 0.932274 | -10.153595 |
| 14 | C | 1.142059 | 3.206048 | -10.512629 |
| 15 | C | 0.933575 | 2.083656 | -8.340017 |
| 16 | C | 1.115721 | 0.964552 | -7.481048 |
| 17 | C | 0.570889 | 0.936825 | -6.20431 |
| 18 | C | -0.197903 | 2.006638 | -5.692588 |
| 19 | C | -0.386964 | 3.107852 | -6.555138 |
| 20 | C | 0.150456 | 3.154692 | -7.836802 |
| 21 | C | -2.503498 | -1.535563 | 3.349275 |
| 22 | N | -3.061436 | -1.494679 | 4.558197 |
| 23 | C | -3.739043 | -0.376266 | 4.921826 |
| 24 | C | -3.849907 | 0.703368 | 3.968149 |
| 25 | N | -3.262901 | 0.640201 | 2.774629 |
| 26 | C | -4.936841 | -1.129979 | 6.826104 |
| 27 | O | -5.575528 | -2.082764 | 6.290799 |
| 28 | C | -5.094236 | -0.926228 | 8.330819 |
| 29 | H | -4.523295 | -0.067339 | 8.69484 |
| 30 | H | -6.153677 | -0.784886 | 8.579623 |
| 31 | H | -4.758082 | -1.82806 | 8.857963 |
| 32 | C | -4.603818 | 1.954477 | 4.264292 |
| 33 | C | -4.071332 | 3.196024 | 3.872028 |
| 34 | C | -5.865246 | 1.933901 | 4.886081 |
| 35 | C | -4.771379 | 4.383368 | 4.102845 |
| 36 | H | -3.099009 | 3.224064 | 3.388671 |
| 37 | C | -6.572225 | 3.119409 | 5.104842 |
| 38 | H | -6.297181 | 0.986186 | 5.189373 |
| 39 | C | -6.02701 | 4.349826 | 4.71968 |
| 40 | H | -4.336087 | 5.333118 | 3.801563 |
| 41 | H | -7.55122 | 3.081627 | 5.576416 |
| 42 | H | -6.575101 | 5.27158 | 4.897907 |
| 43 | N | -4.176492 | -0.223265 | 6.205291 |
| 44 | C | -2.572089 | -0.473321 | 2.432426 |
| 45 | C | -1.926197 | -0.568494 | 1.130588 |
| 46 | H | -1.377495 | -1.492993 | 0.94772 |
| 47 | C | -1.960169 | 0.376226 | 0.151432 |
| 48 | H | -2.514452 | 1.29623 | 0.335554 |
| 49 | C | -1.312197 | 0.241976 | -1.126753 |
| 50 | H | -0.753101 | -0.677968 | -1.309281 |
| 51 | C | -1.360506 | 1.186966 | -2.111858 |
| 52 | H | -1.922295 | 2.104433 | -1.924309 |
| 53 | C | -0.723352 | 1.066619 | -3.397041 |
| 54 | H | -0.163176 | 0.148929 | -3.578343 |
| 55 | C | -0.794394 | 2.024716 | -4.363684 |
| 56 | H | -1.366515 | 2.924952 | -4.131627 |

- 1. **TD DFT calculations for excitations to the first three singlet-excited states of the compounds using B3LYP/6-31+G(d) (IEF-PCM, DMSO)..**

**4.5.1 Excitation of oxy-1a’.**

Excitation energies and oscillator strengths:

Excited State 1: Singlet-A 3.1403 eV 394.82 nm f=0.4328

92 -> 93 0.68946

92 -> 94 -0.14343

Excited State 2: Singlet-A 3.6038 eV 344.04 nm f=0.4462

92 -> 93 0.14238

92 -> 94 0.68710

Excited State 3: Singlet-A 4.0176 eV 308.61 nm f=0.0015

86 -> 93 -0.11656

87 -> 93 0.22992

88 -> 93 -0.17109

89 -> 93 0.27662

90 -> 93 0.53392

91 -> 93 -0.15830

**4.5.2 Excitation of oxy-1b’.**

Excitation energies and oscillator strengths:

Excited State 1: Singlet-A 2.7569 eV 449.72 nm f=0.9552

99 ->100 0.70116

Excited State 2: Singlet-A 3.4822 eV 356.05 nm f=0.3455

99 ->101 0.69857

Excited State 3: Singlet-A 3.8179 eV 324.75 nm f=0.0088

96 ->100 0.58173

97 ->100 0.16191

98 ->100 -0.30925

**4.5.3 Excitation of oxy-1c’.**

Excitation energies and oscillator strengths:

Excited State 1: Singlet-A 2.5551 eV 485.24 nm f=1.4202

106 ->107 0.70286

Excited State 2: Singlet-A 3.3127 eV 374.27 nm f=0.3183

106 ->108 0.69777

Excited State 3: Singlet-A 3.6534 eV 339.37 nm f=0.2037

103 ->107 -0.23860

105 ->107 0.61196

106 ->109 0.21291

**4.5.4 Excitation of oxy-1d’.**

Excitation energies and oscillator strengths:

Excited State 1: Singlet-A 2.3650 eV 524.25 nm f=1.8489

113 ->114 0.70356

Excited State 2: Singlet-A 3.1808 eV 389.79 nm f=0.3610

113 ->115 0.69557

Excited State 3: Singlet-A 3.3623 eV 368.75 nm f=0.2902

112 ->114 0.65216

113 ->116 -0.23486

**4.5.5 Excitation of oxy-1a’(an).**

Excitation energies and oscillator strengths:

Excited State 1: Singlet-A 3.3319 eV 372.11 nm f=0.5083

92 -> 93 0.68417

92 -> 94 0.11712

Excited State 2: Singlet-A 3.7178 eV 333.49 nm f=0.4716

91 -> 93 -0.11167

92 -> 94 0.67770

Excited State 3: Singlet-A 3.7957 eV 326.64 nm f=0.0778

88 -> 93 -0.14311

90 -> 93 -0.38376

91 -> 93 0.53332

92 -> 93 -0.12072

92 -> 94 0.10420

**4.5.6 Excitation of oxy-1b’(an).**

Excitation energies and oscillator strengths:

Excited State 1: Singlet-A 2.9063 eV 426.61 nm f=1.2109

99 ->100 0.69743

Excited State 2: Singlet-A 3.5211 eV 352.12 nm f=0.0092

95 ->100 0.10408

97 ->100 0.51910

97 ->101 0.10889

98 ->100 -0.42741

Excited State 3: Singlet-A 3.6163 eV 342.85 nm f=0.3494

99 ->101 0.69012

**4.5.7 Excitation of oxy-1c’(an).**

Excitation energies and oscillator strengths:

Excited State 1: Singlet-A 2.6991 eV 459.36 nm f=1.8405

106 ->107 0.70013

Excited State 2: Singlet-A 3.3718 eV 367.71 nm f=0.0128

104 ->107 0.39918

104 ->108 -0.11272

105 ->107 0.48341

106 ->108 -0.27183

Excited State 3: Singlet-A 3.4201 eV 362.52 nm f=0.2262

104 ->107 0.25390

105 ->107 0.10152

106 ->108 0.62478

**4.5.8 Excitation of oxy-1d’(an).**

Excitation energies and oscillator strengths:

Excited State 1: Singlet-A 2.4933 eV 497.27 nm f=2.3909

113 ->114 0.70235

Excited State 2: Singlet-A 3.1619 eV 392.11 nm f=0.0079

111 ->114 -0.16443

112 ->114 0.62566

113 ->115 -0.24134

Excited State 3: Singlet-A 3.3253 eV 372.85 nm f=0.1184

111 ->114 -0.42601

111 ->115 -0.13122

113 ->115 0.51921

**4.5.9 Excitation of oxy-2a’.**

Excitation energies and oscillator strengths:

Excited State 1: Singlet-A 3.0003 eV 413.24 nm f=0.2184

88 -> 89 0.69375

88 -> 90 0.11953

Excited State 2: Singlet-A 3.3705 eV 367.85 nm f=0.6823

88 -> 89 -0.11852

88 -> 90 0.69338

Excited State 3: Singlet-A 3.9667 eV 312.56 nm f=0.0076

82 -> 89 0.14199

86 -> 89 0.61671

87 -> 89 0.28667

**4.5.10 Excitation of oxy-2b’.**

Excitation energies and oscillator strengths:

Excited State 1: Singlet-A 2.7000 eV 459.20 nm f=0.8590

95 -> 96 0.69373

95 -> 97 -0.11806

Excited State 2: Singlet-A 3.1616 eV 392.16 nm f=0.4689

95 -> 96 0.11970

95 -> 97 0.69378

Excited State 3: Singlet-A 3.7890 eV 327.22 nm f=0.0627

93 -> 96 0.45586

94 -> 96 0.49424

**4.5.11 Excitation of oxy-2c’.**

Excitation energies and oscillator strengths:

Excited State 1: Singlet-A 2.5189 eV 492.22 nm f=1.3679

102 ->103 0.69755

Excited State 2: Singlet-A 3.0002 eV 413.26 nm f=0.3741

102 ->104 0.69577

Excited State 3: Singlet-A 3.5635 eV 347.93 nm f=0.2697

100 ->103 0.12363

101 ->103 0.63225

102 ->105 -0.24767

**4.5.12 Excitation of oxy-2d’.**

Excitation energies and oscillator strengths:

Excited State 1: Singlet-A 2.3386 eV 530.17 nm f=1.8423

109 ->110 0.70067

Excited State 2: Singlet-A 2.8861 eV 429.60 nm f=0.3033

109 ->111 0.69677

Excited State 3: Singlet-A 3.2993 eV 375.79 nm f=0.3958

108 ->110 0.63128

109 ->112 0.27302

**4.5.13 Excitation of oxy-2a’(an).**

Excitation energies and oscillator strengths:

Excited State 1: Singlet-A 3.0746 eV 403.26 nm f=0.1886

87 -> 89 -0.10192

88 -> 89 0.69595

Excited State 2: Singlet-A 3.5350 eV 350.73 nm f=0.3909

86 -> 89 0.19151

87 -> 89 -0.35905

87 -> 90 -0.11363

88 -> 90 0.54467

Excited State 3: Singlet-A 3.5832 eV 346.01 nm f=0.4072

84 -> 89 -0.12813

86 -> 89 -0.19004

87 -> 89 0.50056

88 -> 90 0.41900

**4.5.14 Excitation of oxy-2b’(an).**

Excitation energies and oscillator strengths:

Excited State 1: Singlet-A 2.7719 eV 447.29 nm f=0.8361

95 -> 96 0.68885

95 -> 97 0.12450

Excited State 2: Singlet-A 3.2403 eV 382.63 nm f=0.7119

95 -> 96 -0.12600

95 -> 97 0.68479

Excited State 3: Singlet-A 3.3980 eV 364.87 nm f=0.0348

91 -> 96 0.10783

93 -> 96 0.40021

94 -> 96 0.53727

**4.5.15 Excitation of oxy-2c’(an).**

Excitation energies and oscillator strengths:

Excited State 1: Singlet-A 2.6239 eV 472.53 nm f=1.5368

102 ->103 0.68861

102 ->104 0.13110

Excited State 2: Singlet-A 3.0767 eV 402.98 nm f=0.5647

102 ->103 -0.13551

102 ->104 0.68398

Excited State 3: Singlet-A 3.2740 eV 378.69 nm f=0.0236

100 ->103 -0.32189

101 ->103 0.58836

**4.5.16 Excitation of oxy-2d’(an).**

Excitation energies and oscillator strengths:

Excited State 1: Singlet-A 2.4530 eV 505.44 nm f=2.2081

109 ->110 0.69613

Excited State 2: Singlet-A 3.0006 eV 413.20 nm f=0.3597

108 ->111 0.10271

109 ->111 0.68760

Excited State 3: Singlet-A 3.0886 eV 401.42 nm f=0.0212

107 ->110 -0.18263

108 ->110 0.65033

109 ->112 0.12938

**References**

1. R. Nishihara, E. Hoshino, Y. Kakudate, S. Kishigami, N. Iwasawa, S. Sasaki, T. Nakajima, M. Sato, S. Nishiyama, D. Citterio, K. Suzuki and S. B. Kim, *Bioconjugate Chem.*, 2018, **29**, 1922-1931.

2. S. B. Kim, S. Miller, N. Suzuki, T. Senda, R. Nishihara and K. Suzuki, *Anal. Sci.*, 2015, **31**, 955-960.

3. S. B. Kim, M. Torimura and H. Tao, *Bioconjugate Chem.*, 2013, **24**, 2067–2075.

4. R. Nishihara, R. Paulmurugan, T. Nakajima, E. Yamamoto, A. Natarajan, R. Afjei, Y. Hiruta, N. Iwasawa, S. Nishiyama, D. Citterio, M. Sato, S. B. Kim and K. Suzuki, *Theranostics*, 2019, **9**, 2646-2661.

5. M. J. Frisch, G. W. Trucks, H. B. Schlegel, G. E. Scuseria, M. A. Robb, J. R. Cheeseman, G. Scalmani, V. Barone, B. Mennucci, G. A. Petersson, H. Nakatsuji, M. Caricato, X. Li, H. P. Hratchian, A. F. Izmaylov, J. Bloino, G. Zheng, J. L. Sonnenberg, M. Hada, M. Ehara, K. Toyota, R. Fukuda, J. Hasegawa, M. Ishida, T. Nakajima, Y. Honda, O. Kitao, H. Nakai, T. Vreven, J. A. Montgomery, Jr., J. E. Peralta, F. Ogliaro, M. Bearpark, J. J. Heyd, E. Brothers, K. N. Kudin, V. N. Staroverov, R. Kobayashi, J. Normand, K. Raghavachari, A. Rendell, J. C. Burant, S. S. Iyengar, J. Tomasi, M. Cossi, N. Rega, J. M. Millam, M. Klene, J. E. Knox, J. B. Cross, V. Bakken, C. Adamo, J. Jaramillo, R. Gomperts, R. E. Stratmann, O. Yazyev, A. J. Austin, R. Cammi, C. Pomelli, J. W. Ochterski, R. L. Martin, K. Morokuma, V. G. Zakrzewski, G. A. Voth, P. Salvador, J. J. Dannenberg, S. Dapprich, A. D. Daniels, Ö. Farkas, J. B. Foresman, J. V. Ortiz, J. Cioslowski and D. J. Fox, *Gaussian 09, Revision D.01*, Gaussian, Inc.: Wallingford, CT, 2004.

6. A. D. Becke, *J. Chem. Phys*., 1993, **98**, 5648-5652.

7. C. T. Lee, W. T. Yang and R. G. Parr, *Phys. Rev. B*, 1988, **37**, 785-789.

8. P. J. Stephens, F. J. Devlin, C. F. Chabalowski and M. J. Frisch, *J. Phys. Chem*., 1994, **98**, 11623-11627.

9. J. Tomasi, B. Mennucci and R. Cammi, *Chem. Rev*., 2005, **105**, 2999-3094.

10. R. Dennington, T. Keith and J. Millam, *GaussView*, Version 5. Semichem Inc., Shawnee Mission KS, 2009.
